# Impact of Skin Pigmentation on Pulse Oximetry Blood Oxygenation and Wearable Pulse Rate Accuracy: Systematic Review and Meta-Analysis

**DOI:** 10.2196/62769

**Published:** 2024-10-10

**Authors:** Sanidhya Singh, Miles Romney Bennett, Chen Chen, Sooyoon Shin, Hamid Ghanbari, Benjamin W Nelson

**Affiliations:** 1 University of Michigan Medical School Ann Arbor, MI United States; 2 Verily Life Sciences LLC South San Francisco, CA United States; 3 Division of Digital Psychiatry Department of Psychiatry Beth Israel Deaconess Medical Center, Harvard Medical School Boston, MA United States

**Keywords:** photoplethysmography, pulse oximetry, arterial blood gas, skin tone, skin pigmentation, bias, digital technology

## Abstract

**Background:**

Photoplethysmography (PPG) is a technology routinely used in clinical practice to assess blood oxygenation (SpO_2_) and pulse rate (PR). Skin pigmentation may influence accuracy, leading to health outcomes disparities.

**Objective:**

This systematic review and meta-analysis primarily aimed to evaluate the accuracy of PPG-derived SpO_2_ and PR by skin pigmentation. Secondarily, we aimed to evaluate statistical biases and the clinical relevance of PPG-derived SpO_2_ and PR according to skin pigmentation.

**Methods:**

We identified 23 pulse oximetry studies (n=59,684; 197,353 paired SpO_2_-arterial blood observations) and 4 wearable PR studies (n=176; 140,771 paired PPG-electrocardiography observations). We evaluated accuracy according to skin pigmentation group by comparing SpO_2_ accuracy root-mean-square values to the regulatory threshold of 3% and PR 95% limits of agreement values to +5 or –5 beats per minute (bpm), according to the standards of the American National Standards Institute, Association for the Advancement of Medical Instrumentation, and the International Electrotechnical Commission. We evaluated biases and clinical relevance using mean bias and 95% CI.

**Results:**

For SpO_2_, accuracy root-mean-square values were 3.96%, 4.71%, and 4.15%, and pooled mean biases were 0.70% (95% CI 0.17%-1.22%), 0.27% (95% CI –0.64% to 1.19%), and 1.27% (95% CI 0.58%-1.95%) for light, medium, and dark pigmentation, respectively. For PR, 95% limits of agreement values were from –16.02 to 13.54, from –18.62 to 16.84, and from –33.69 to 32.54, and pooled mean biases were –1.24 (95% CI –5.31 to 2.83) bpm, –0.89 (95% CI –3.70 to 1.93) bpm, and –0.57 (95% CI –9.44 to 8.29) bpm for light, medium, and dark pigmentation, respectively.

**Conclusions:**

SpO_2_ and PR measurements may be inaccurate across all skin pigmentation groups, breaching U.S. Food and Drug Administration guidance and industry standard thresholds. Pulse oximeters significantly overestimate SpO_2_ for both light and dark skin pigmentation, but this overestimation may not be clinically relevant. PRs obtained from wearables exhibit no statistically or clinically significant bias based on skin pigmentation.

## Introduction

Photoplethysmography (PPG) technology has been used in medicine since the 1970s to assess pulse rate (PR) and blood oxygenation (SpO_2_). The accuracy of PPG-based SpO_2_ and PR is critical for medical practice, clinical decision-making, and patient outcomes [1].

Technological advancements have led to rapid expansion of this technology into consumer devices [2]. This has enabled consumers to continuously and unobtrusively track health status, generating information that is becoming commonly used in health care settings and as a source of research end points [3,4].

Researchers have identified several factors that influence the accuracy of PPG-based measurements, including skin temperature, sensor contact pressure, skin thickness, and hydration level [5,6]. Notably, darker skin pigmentation may influence PPG-based SpO_2_ and PR readings [7-10]. Patients with darker skin pigmentation are more likely than patients with lighter skin color to have overestimated SpO_2_ readings, leading to lower hospital admission; higher occult hypoxemia; and delayed or no access to dexamethasone, therapeutic oxygen, and COVID-19 therapies, resulting in increased hospital readmission, organ dysfunction, and mortality [11-18].

Starting in 2013, a series of U.S. Food and Drug Administration (FDA) guidances, safety communications, and more recently, statements from attorneys general have called to address darker skin pigmentation bias in pulse oximeters [19]; however, these guidances lacked standards for assessing skin tone [20]. Recent pulse oximeter research has rekindled interest in skin pigmentation disparities in PPG sensor accuracy, leading to increased media and regulatory attention [21-24]. In November 2023, more than 24 attorneys general wrote a letter calling on the FDA to take urgent action to address pulse oximeter skin pigmentation disparities [25,26].

More recent research has also highlighted the potential presence of these biases in research-grade and consumer PPG-based wearable devices [4,27-29], leading to calls to address inequity, bias, and discrimination in wearable health technology and clinical practice algorithms [30,31].

Accuracy guidance for pulse oximeters and heart rate or PR devices has been delineated by regulatory bodies, industry, and medical standards. Overall accuracy of pulse oximeters can be assessed with accuracy root-mean-square (A_rms_), which combines mean bias and precision (SD of bias) into a single metric [32]. FDA guidance has a threshold of A_rms_≤3% [19] for transmittance devices and a threshold of A_rms_≤3.5% [19] for ear clip and reflectance devices, while international thresholds are set at A_rms_≤4% [32]. For heart rate or PR devices, overall accuracy can be assessed with 95% limits of agreement (LoAs). The American National Standards Institute (ANSI), Association for the Advancement of Medical Instrumentation (AAMI), and International Electrotechnical Commission (IEC) have set the recommendation that electrocardiography (ECG) devices have mean bias of +5 or –5 beats per minute (bpm) or mean absolute percentage error≤10%, whichever is greater [33,34]. Devices with accuracy measures breaching these accuracy thresholds can produce questionable results.

It is, therefore, critical to generate evidence to inform the design and calibration of these devices to reduce algorithmic bias and improve accuracy, so that PPG-based technology can generalize to all segments of the population, mitigating racial disparities in health outcomes. There have been systematic reviews on PPG skin pigmentation bias, but these have been limited to either consumer device PR [35] or pulse oximeter SpO_2_ [36-38]. Only 1 meta-analysis focused on pulse oximetry for this topic is available [36], and an additional 7 studies have been published recently, providing an additional 50,980 participants and 182,369 paired observations. Therefore, performing a comprehensive meta-analysis to examine PPG accuracy, potential bias, and clinical relevance for SpO_2_ and PR by skin pigmentation is timely.

## Methods

### Search Strategy and Selection Criteria

We followed the PRISMA (Preferred Reporting Items for Systematic Reviews and Meta-Analyses) guidelines [39] and developed a MEDLINE systematic search. Searches were performed between April 2022 and June 2023, and a final ad hoc search was conducted in June 2023. No additional studies were included after this date.

We included studies that (1) investigated PPG-derived SpO_2_ or PR test devices per definition [40]; (2) used arterial blood gas (SaO_2_) or ECG as the reference device; and (3) reported mean bias and SD, SE, 95% LoA, or 95% CI by race, ethnicity, or skin tone. Inclusion disagreements were resolved by consensus.

Exclusion criteria included (1) literature reviews, systematic reviews, commentary, and meta-analyses; (2) non-English manuscripts; (3) irretrievable full source texts; (4) studies on remote PPG; and (5) those that lack SaO_2_ or ECG as the reference device. We chose to not exclude papers based on measurement hardware or underlying algorithms given that all measurement devices relied on contact-based PPG to measure the same end points of interest—SpO_2_ and PR.

### Data Analysis

#### Data Extraction

**Figure 1:**
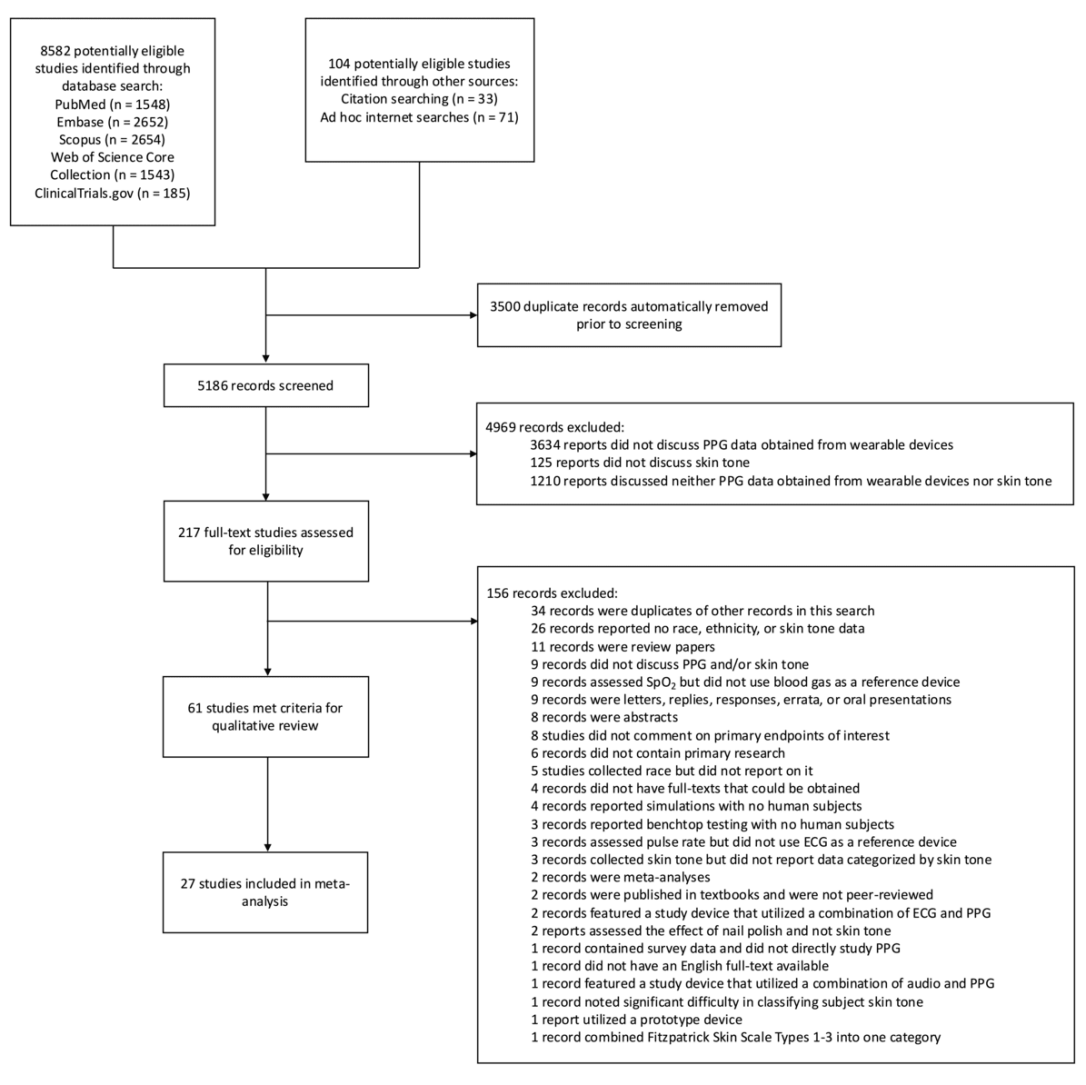
PRISMA (Preferred Reporting Items for Systematic Reviews and Meta-Analyses) diagram. ECG: electrocardiography; PPG: photoplethysmography; SpO_2_: blood oxygenation.

Data were independently extracted from published manuscripts (BWN and SS). A third and fourth reviewer (MRB and HG) adjudicated any differences between the initial 2 reviewers and resolved any disagreements by checking manuscripts. The first author verified the quality of manuscripts. Standardized data extraction was developed to extract the study characteristics (see the Materials—Data Extraction section in Multimedia Appendix 1). For 1 paper, we used participant-level data from the supplementary materials to calculate mean bias and SE by skin pigmentation category [41]. This resulted in 27 studies in our final analysis (Figure 1 and Table 1).

**Table 1:** Characteristics of included studies.

Device type and study (author, year)			Sample size, n		Test device evaluated		Reference device		SaO_2_^a^ (%) range		Participant population		Research setting		Skin pigmentation method
**Pulse Oximetry**															
	Abrams et al [42], 2003	200		Nellcor N-200		Radiometer ABL-520		Not reported		Medical—adult patients with cirrhosis		Inpatient		Race—Black and White	
	Adler et al [43], 1998	298		Nellcor D-25		4-wavelength spectro-photometer, or co-oximeter (Radiometer OSM3)		50-99		Medical—adult emergency department patients		Inpatient		Skin tone—Munsell color system categorized into light, medium, or dark	
	Andrist et al [44], 2022	1061		Not reported		Not reported		Not reported		Medical—pediatric		Inpatient		Race	
	Barker and Wilson [45], 2023	75		Masimo SET^b^ pulse oximeters with RD-SET sensors		Radiometer ABL-835 Flex CO-Oximeter		70-100		Mixed—healthy and mild systemic disease		Research — laboratory		Race	
	Bickler et al [10], 2005	23		Nellcor N-595 with Nellcor OxiMax A finger probe Novametrix 513s models (2 types) Nonin Onyx models (2 types)		Radiometer OSM3		60-100		Healthy—nonsmoking		Research — laboratory		Ethnicity—light (Northern European) and dark (African American)	
	Bothma et al [46], 1996	100		Simed S100e Nihon Koden Ohmeda 3740		IL482 Co-oximeter System		87.8-99.2		Medical—critically ill adult patients		Inpatient		Skin tone—EEL^c^ reflectance spectrophotometer; all participants had dark skin tone	
	Burnett et al [47], 2022	46,253		Unspecified Nellcor and Masimo devices		GEMStat Premier 3000		Not reported		Medical—patients receiving anesthesia		Inpatient		Race and ethnicity	
	Crooks et al [48], 2022	2997		Not reported		Not reported		Not reported		Medical—COVID-19		Inpatient		Race	
	Ebmeier et al [49], 2018	394		Marquette Rac-4A monitors with Masimo sensors and Philips IntelliVue MP70 monitors with Philips Adult Reusable SpO_2_^d^ sensors		Radiometer ABL 800 FLEX SaO_2_ analyzer		Not reported		Medical—multiple conditions		Inpatient		Ethnicity	
	Fawzy et al [17], 2022	1216		Not reported		ABL825, ABL827, or ABL90 blood gas analyzers		Not reported		Medical—COVID-19		Inpatient		Race and ethnicity	
	Feiner et al [8], 2007	36		Nellcor N-595 (OxiMax A adhesive probe) Nellcor N-595 (a clip-type probe) Masimo Radical (clip probe) Masimo Radical (adhesive disposable probe) Nonin 9700 (clip-type probe) Nonin 9700 (disposable adhesive probe)		Radiometer OSM3 multiwavelength oximeter		60-100		Healthy—nonsmoking		Research — laboratory		Ethnicity—light (Caucasian), intermediate (Hispanic, Indian, Filipino, or Vietnamese), and dark (African American) categories	
	Foglia et al [50], 2017	36		Nellcor Oximax (Covidien) Masimo Rainbow SET Radical 7		Siemens Rapidlab 1265		60-92		Medical—infants with cyanotic congenital heart disease and oxygen saturation <90%		Inpatient		Skin tone—Munsell color system	
	Jubran and Tobin [51], 1990	54		Nellcor pulse oximeter with disposable or reusable probes Ohmeda-Biox3700 pulse oximeter with reusable probe		CO-oximetry		Not reported		Medical—critically ill, ventilator-dependent patients		Inpatient		Race—Black and White	
	McGovern et al [52], 1996	8		Ohmeda 3700		IL 482 Co-oximeter		Not reported		Medical—adults with stable condition with severe COPD^f^		Research — laboratory		Race—all White	
	Muñoz et al [53], 2008	846		Minolta Pulsox-7		IL 682 co-oximeter		Not reported		Medical—adults under assessment for long-term home oxygen therapy		Outpatient		Race—all White	
	Pilcher et al [54], 2020	400		Carescape B450 monitor with Nellcor probe GE Dash 3000 Masimo Radical 7 Masimo SET Quartz (unspecified) Masimo SET Quartz Q400 Nonin 2120 Nonin 2140 Nonin Avant (unspecified) Nonin Avant 4000 Nonin Avant 9700 Nonin Lifesense Medair Novametrix Model 512 Ohmeda Biox 3700E with a GE TruSignal or Nellcor probe Philips Intellivue MP70 with a GE TruSignal Nellcor or Philips probe Welch Allyn with a Nellcor probe		Radiometer ABL800		72-100		Medical—hospitalized adult patients		Inpatient and outpatient		Skin Tone—Fitzpatrick Scale categorized into light, medium, and dark	
	Ruppel et al [55], 2023	774		Not reported		Not reported		Not reported		Medical—cardiac catheterization		Inpatient		Race	
	Sudat et al [56], 2023	8735		Not reported		Not reported		Not reported		Medical—multiple conditions		Inpatient		Race	
	Thrush and Hodges [57], 1994	25		Critikon Dinamap Plus Model 8700 Critikon Oxyshuttle Ohmeda 3700 Catalyst Research MiniOx IV		IL482 co-oximeter		80-100		Healthy—nonsmoking adults		Research — laboratory		Race—all White	
	Valbuena et al [12], 2022	372		Not reported		Not reported, blood gas analysis		Not reported		Medical—adult patients with respiratory failure or COVID-19		Inpatient		Race and ethnicity—White, Black, Hispanic, and Asian	
	Vesoulis et al [58], 2021	294		Nellcor SpO_2_ module with Neonatal-Adult MAX-N adhesive SpO_2_ sensor (Covidien) (used with either Philips IntelliVue MP70 or MX800 monitors)		Radiometer ABL800 Flex		Not reported		Medical—preterm infants at neonatal intensive care unit		Inpatient		Race—White and Black	
	Wiles et al [59], 2022	194		Nellcor reusable SpO_2_ probes or Mindray disposable SpO_2_ probes (GE Healthcare B1x5 M/P monitor)		RAPIDpoint 500 analyser (Siemens Healthcare GmbH)		Not reported		Medical—adult patients with COVID-19 pneumonitis		Inpatient		Race—Asian, Black, White, and other	
	Zeballos and Weisman [9], 1991	33		Hewlett-Packard HP-47201A Ohmeda Biox IIA		IL282 co-oximeter		Not reported		Healthy—non-smoking volunteers		Research — laboratory		Race—all Black	
**Pulse Rate**															
	Bent et al [41], 2020	53		Empatica E4 Apple Watch Fitbit Charge Garmin Vivosmart 3 Xiaomi Miband Biovotion Everion		BittiumFaros 180, Bittium Inc.		N/A^g^		Healthy		Research — laboratory		Skin tone—Fitzpatrick Scale: 1 (n=7) 2 (n=8) 3 (n=10) 4 (n=9) 5 (n=9) 6 (n=10)	
	Nelson and Allen [60], 2019	1		Apple Watch 3 Fitbit Charge 2		Vrije Universiteit Ambulatory Monitoring System		N/A		Healthy		Research — real world		Skin tone—Fitzpatrick Scale: 1 (n=1)	
	Sanudo et al [61], 2019	45		Apple Watch (version not reported)		Polar Chest Strap		N/A		Healthy		Research — laboratory		Skin tone—Fitzpatrick Scale: 2 (n=15) 3 (n=15) 4 (n=15)	
	Chow and Yang [62], 2020	40		Garmin Vivosmart HR+ Xiaomi Mi Band 2		Polar H7 Chest Strap		N/A		Healthy		Research — laboratory		Ethnicity and skin tone—East Asian, Fitzpatrick Scale 3 and 4	

^a^SaO_2_: arterial blood gas.

^b^SET: Signal Extraction Technology.

^c^EEL: electron energy loss.

^d^SpO_2_: blood oxygenation.

^f^COPD: chronic obstructive pulmonary disease.

^g^N/A: not applicable.

#### Quality Assessment of the Overall Evidence

QUADAS-2 tool was used to evaluate risk of bias and applicability (Figure S1 in Multimedia Appendix 1). Funnel plots evaluated publication bias using the *metafor* package [63] (Figures S2 and S3 in Multimedia Appendix 1).

### Statistical Analysis

#### Skin Tone Categorization

We mapped skin tone, race, and ethnicity into 3 primary skin pigmentation groups of light, medium, and dark, following published methodology [36] (see Table S1 in Multimedia Appendix 1) and examined biases for SpO_2_ and PR by each skin pigmentation group (described below).

We elected to use this same skin pigmentation categorization schema as has previously been used, with the goal of expanding upon the analysis of Shi et al [36].

#### Statistical Analysis

The objective of the study was to assess whether the devices were accurate in estimating SpO_2_ and PR when compared with a SaO_2_ and ECG reference device, respectively, for each skin pigmentation group. If these measures were found to be inaccurate, biases were quantified and their clinical relevance was assessed. The analytical approach to execute these research objectives was formulated based on methodologies used in prior meta-analyses within the discipline [36].

#### Evaluation End Points and Criteria

A summary of the evaluation criteria used can be found in Table 2.

**Table 2:** Evaluation end points and criteria for blood oxygenation (SpO_2_) and pulse rate studies.

Category and objective		Evaluation end point	Evaluation criteria
**SpO_2_**			
	Evaluate accuracy	A_rms_^a^	Accurate if A_rms_≤3%
	Statistically significant bias	Mean bias (95% CI)	Statistically significant bias if 95% CI does not contain 0
	Clinically relevant bias	Mean bias (95% CI)	Clinically relevant bias if either upper bound of 95% CI≤4% or lower bound of 95% CI>4%
**Pulse rate**			
	Evaluate accuracy	95% LoA^b^	Accurate if 95% LoA is bounded by +5 or –5 bpm^c^
	Statistically significant bias	Mean bias (95% CI)	Statistically significant bias if 95% CI does not contain 0
	Clinically relevant bias	Mean bias (95% CI)	Clinically relevant bias if either upper bound of 95% CI ≤5 bpm or lower bound of 95% CI >5 bpm

^a^A_rms_: accuracy root-mean-square.

^b^LoA: limits of agreement.

^c^bpm: beats per minute.

#### Accuracy

To evaluate the accuracy of SpO_2_ , A_rms_ was used to measure accuracy, because of its commonality in the regulatory space [19]. It was calculated as 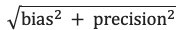, where precision is the SD of bias [42,43]. A_rms_ is useful in clinical settings because it provides a single metric that accounts for both the systematic error (bias) and the random error (precision) in measurements and it provides a comprehensive assessment of how close the measurements are to the true values. A lower A_rms_ value indicates better accuracy, meaning that the measurement is closer to the true value, whereas a higher A_rms_ value suggests lower accuracy, indicating that the measurement is further away from the true value.

For SpO_2_, our study used the stricter A_rms_>3% threshold to define inaccuracy as per FDA guidance [19]. We used this threshold as 22 (96%) out of 23 of pulse oximeter studies used a transmittance device and only one used an ear clip, which would have set a more liberal threshold of 3.5%. To evaluate accuracy of PR, the 95% LoA of bias was used, and LoA was constructed as mean bias (SD 1.96). For PR, a pooled 95% LoA not bounded by +5 or –5 bpm was considered inaccurate per ANSI/AAMI/IEC standards [33,34].

#### Bias

To evaluate bias of both SpO_2_ and PR, mean bias and its 95% CI were used. For each data point that compares the test with the reference device, bias was constructed as *test device – reference device*. Based on the final results, a measure was considered to have statistically significant bias if the 95% CI of its mean bias did not contain 0, with the estimated mean bias over 0 indicating overestimation and under 0 indicating underestimation.

#### Clinical Relevance of Estimated Bias

To evaluate the clinical relevance of the estimated bias for SpO_2_, if the 95% CI of mean bias was out of the +4% to –4% range it was considered clinically relevant. This threshold is inferred from FDA guidance on *Pulse Oximeters—Premarket Notification Submissions [510(k)s]: Guidance for Industry and Food and Drug Administration Staff* [19]. To evaluate the clinical relevance of the estimated bias for PR, if the 95% CI of mean bias was out of the bound of +5 or –5 bpm, it was considered clinically relevant as per ANSI/AAMI/IEC standards [33,34].

#### Statistical Meta-Analysis

To obtain pooled results for above listed end points, we collected sample size, paired observations, mean bias, and SD from various studies. When not available, we either transformed relevant parameters, such as 95% LoA and 95% CI, into these statistics or obtained them by analyzing the raw data.

#### Methods to Pool Measures of Bias

Correlated and hierarchical effect (CHE) models were used to pool mean biases from various studies. Specifically, 3-level hierarchical models were constructed, with Level 1 representing individual data points collected in a study, Level 2 representing potentially multiple comparisons within a research study, and Level 3 representing various studies included in the meta-analysis. Within a study, there may be multiple devices compared using the same participants’ data, and these effect sizes are dependent. To account for this dependence, the CHE model used the robust variance estimation (RVE) method, which allowed us to combine data from single-measure design studies with multiple dependent estimates of effect size, even when the dependence is unknown [64]. To execute the CHE model with RVE, we needed to plug in assumed correlation coefficients for these dependent effect sizes. We used correlation coefficient 0.9 following published methods [36], and small (⍴=0.30) and moderate (⍴=0.60) correlation coefficients were also explored in sensitivity analyses. Additionally, one report [48] had substantially larger SE, so we conducted a separate analysis excluding its results as part of the sensitivity analysis. To evaluate the heterogeneity of the studies, we reported the overall *I*^2^ (percentage of variability in the effect sizes that is not caused by sampling error) and its breakdown of within-study (*I*^2^_Level2_) and between-study (*I*^2^_Level3_) portions. We conducted subgroup analyses to see if there were statistically significant differences in bias between skin pigmentation categories (Table S2 from Multimedia Appendix 1). Forest plots were provided to visualize the mean bias (Figures S2 and S3 from Multimedia Appendix 1).

#### Methods to Pool Measures of Accuracy

When it comes to providing pooled 95% LoA and A_rms_, no established methodologies exist, so we followed an analytical approach used in prior meta-analyses within the discipline [36]. Specifically, SD of bias across studies were pooled using CHE models similar to those used to pool mean biases. The pooled mean bias and pooled SD were then used to provide pooled estimates of 95% LoA and A_rms_. Specifically,

- Overall accuracy A_rms_ = 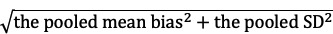
- Overall 95% LoA = pooled mean bias ± 1.96 × pooled SD

#### Other Methods

Descriptive statistics were provided for study characteristics, the device intended use, mean bias based on different skin tones, and reference devices. All analyses were conducted using R (version 4.3.1; R Foundation for Statistical Computing) with statistical models based on R packages *metafor* [63] and *clubSandwich* [65].

## Results

### Study Selection and Characteristics

Search strategy resulted in 8582 records. We selected 27 studies for full review (pulse oximetry: n=23, PR: n=4; Figure 1, Table 1, and Table 2). A total of 23 pulse oximetry studies involving 59,684 participants with 197,353 paired SpO_2_-SaO_2_ observations were included. Additionally, 4 PR studies with 176 participants and 140,771 paired PR-ECG observations were analyzed (Table 3).

**Table 3:** Summary characteristics of included studies^a^.

Item and subitem		Pulse oximetry (n=23), n (%)	Pulse rate (n=4), n (%)
**Participants (pulse oximetry: n=59,684; pulse rate: n=176)**			
	Light skin pigmentation	40,416 (67.72)	31 (17.61)
	Medium skin pigmentation	9967 (16.70)	129 (73.30)
	Dark skin pigmentation	9301 (15.58)	16 (9.09)
**Paired observations** **(pulse oximetry: n=197,353; pulse rate: n=140,771)**			
	Light skin pigmentation	131,008 (66.38)	43,116 (30.60)
	Medium skin pigmentation	32,095 (16.26)	90,733 (64.50)
	Dark skin pigmentation	34,250 (17.35)	6922 (4·92)
**Device type included in study**			
	Medical	23 (100)	0 (0)
	Nonregulated	0 (0)	4 (100)
**Sensor type**			
	Transmittance	22 (96)	0 (0)
	Reflectance	1 (4)	4 (100)
**Patient population**			
	Healthy	4 (17)	3 (75)
	Medical	18 (78)	0 (0)
	Healthy and medical	1 (4)	0 (0)
	Not reported	0 (0)	1 (25)
**Research setting**			
	Medical inpatient	15 (65)	0 (0)
	Medical outpatient	1 (4)	0 (0)
	Medical combined	1 (5)	0 (0)
	Research laboratory	6 (26)	3 (75)
	Research in the real world	0 (0)	1 (25)
**Skin pigmentation method**			
	Skin tone	4 (17)	4 (100)
	Race	13 (57)	0 (0)
	Ethnicity	3 (13)	0 (0)
	Race and ethnicity	3 (13)	0 (0)

^a^Medical device defined as devices that received regulatory clearance for either blood oxygenation (SpO_2_) or pulse rate (PR).

### Descriptive Statistics on Race, Ethnicity, and Skin Tone

#### Pulse Oximetry

Skin pigmentation was classified by race in 13 (57%) out of 23 studies, ethnicity in 3 (13%) studies, skin tone in 4 (17%) studies, and both race and ethnicity in 3 (13%) studies (Table 3). Of the 59,684 patients with 197,353 paired observations, there were a total of 40,416 (67.72%) patients with light skin pigmentation with 131,008 (66.38%) paired observations, 9967 (16.70%) patients with medium skin pigmentation with 32,095 (16.26%) paired observations, and 9301 (15.58%) patients with dark skin pigmentation with 34,250 (17.35%) paired observations.

#### PR Results

Skin pigmentation was classified by race or ethnicity in 0 (0%) out of 4 studies and skin tone in 4 (100%) studies (Table 3). Of the 176 patients with 140,771 paired observations, there were a total of 31 (17.61%) participants with light skin pigmentation with 43,116 (30.60%) paired observations, 129 (73.30%) participants with medium skin pigmentation with 90,733 (64.50%) paired observations, and 16 (9.09%) participants with dark skin pigmentation with 6922 (4.92%) paired observations.

### PPG Accuracy and Bias by Skin Pigmentation

#### Pulse Oximetry

**Figure 2:**
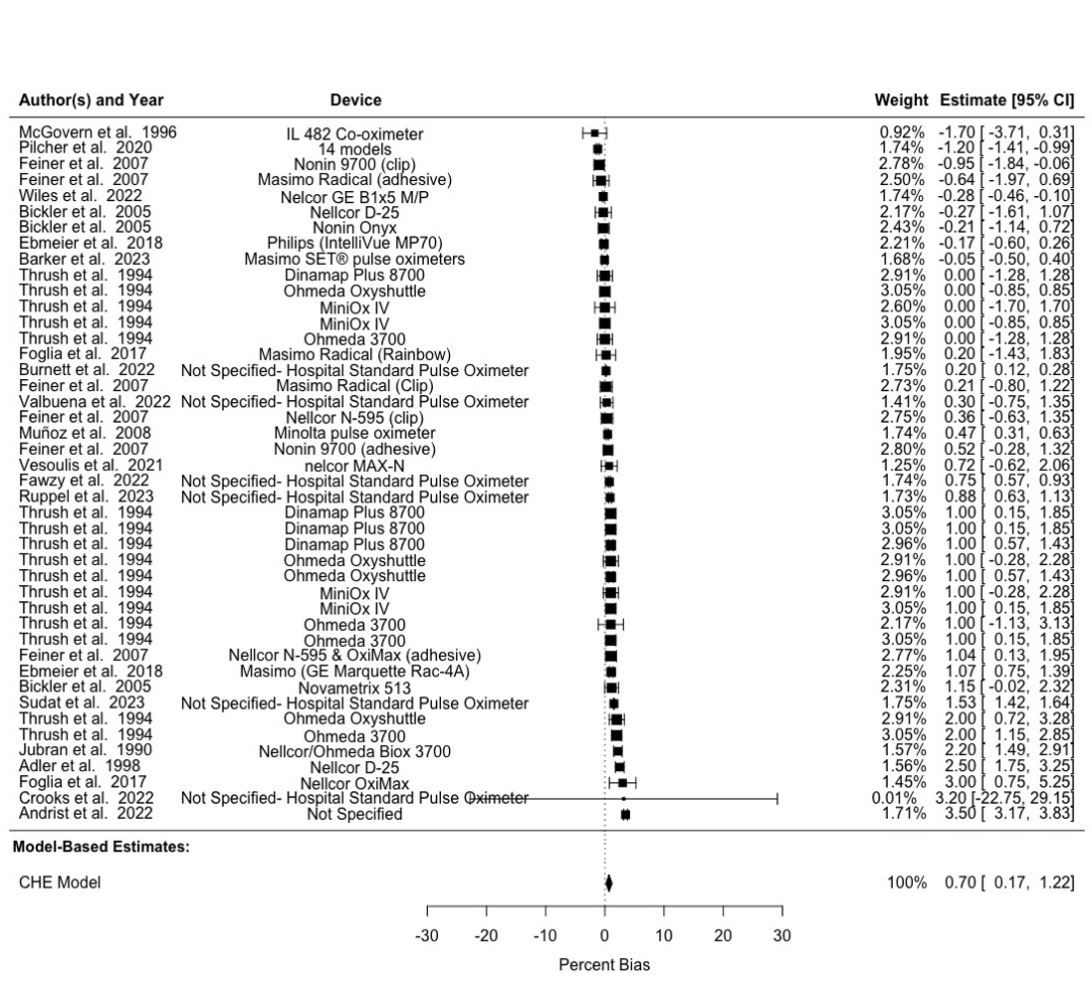
Forest plot showing bias in SpO_2_ measurements in patients with light skin pigmentation. Multiple entries from the same study are included, each representing different devices evaluated. Squares denote study weight; center of squares denote observed study effect size; vertical lines denote study CIs; and diamond denotes pooled effect. CHE: correlated and hierarchical effect; SpO_2_: blood oxygenation.

**Figure 3:**
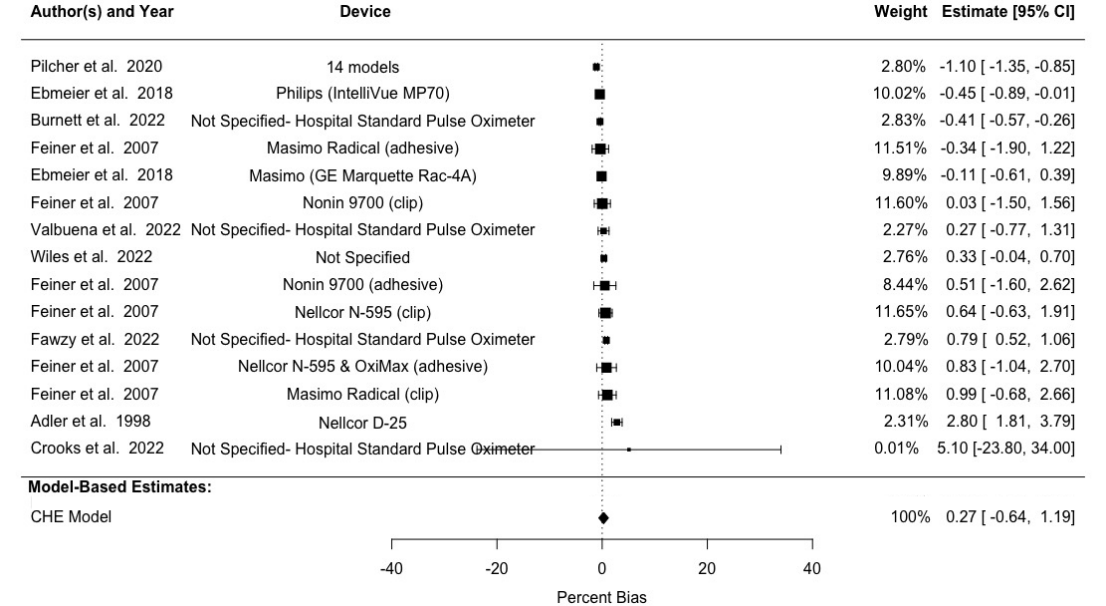
Forest plot showing bias in SpO_2_ measurements in subjects with medium skin pigmentation. Multiple entries from the same study are included, each representing different devices evaluated. Note: squares denote study weight, center of squares denote observed study effect size, vertical lines denote study CIs, and diamond denotes pooled effect. CHE: correlated and hierarchical effect; SpO_2_: blood oxygenation.

**Figure 4:**
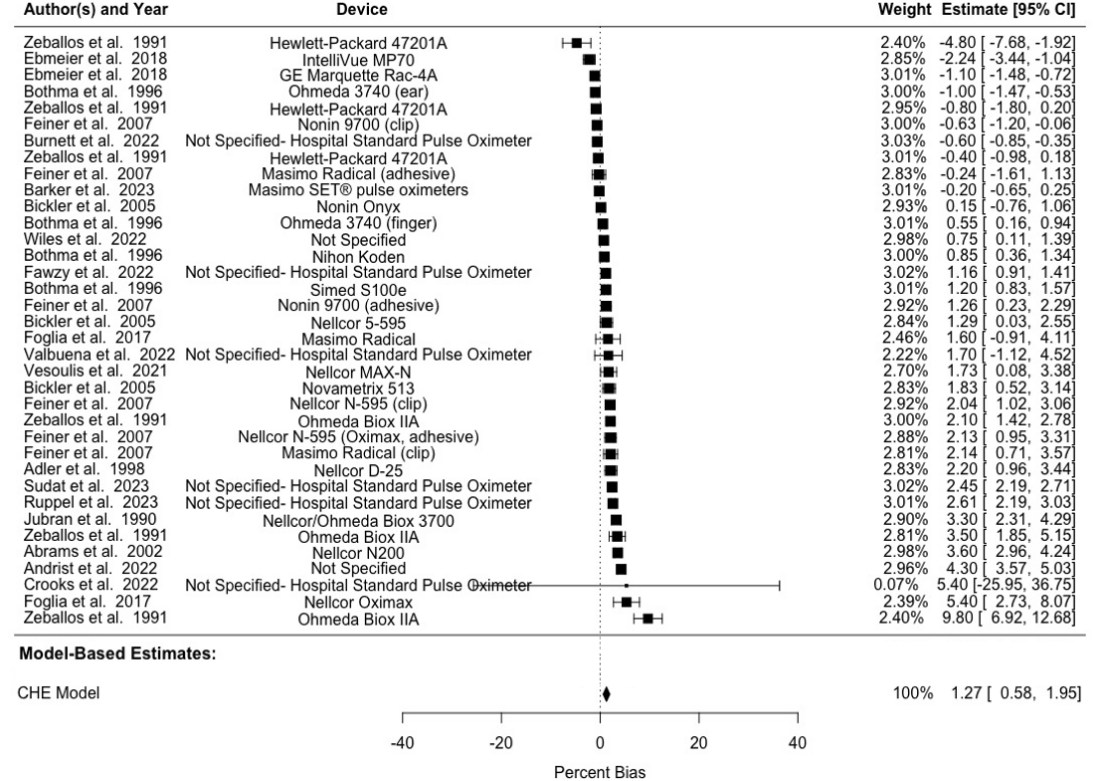
Forest plot showing bias in SpO_2_ measurements in patients with dark skin pigmentation. Multiple entries from the same study are included, each representing different devices evaluated. Squares denote study weight; center of squares denote observed study effect size; vertical lines denote study CIs; and diamond denotes pooled effect. CHE: correlated and hierarchical effect; SpO_2_: blood oxygenation.

The pooled A_rms_ across different skin pigmentation groups was 3.6%, 3.96%, 4.71%, and 4.15% for combined, light, medium, and dark skin pigmentation, respectively (Table 4 and Figures 2-4 [8-11,17,42-51,54-56,58,59]). Of note, studies implementing multiple trial conditions or using multiple study devices were shown multiple times in Figures 2-4 to delineate different devices used within the same study. We observed a pooled mean percent bias of 0.82% (95% CI 0.29%-1.35%) across all skin pigmentation groups using the CHE model. Between-study heterogeneity (*I*^2^_Level3_) accounted for 14.53% of the total variation, while within-study heterogeneity (*I*^2^_Level2_) explained 84.02% of total variation. Delineating by skin pigmentation, the pooled mean percent bias from the CHE model was 0.70% (95% CI 0.17%-1.22%) for light skin, 0.27% (95% CI –0.64% to 1.19%) for medium skin, and 1.27% (95% CI 0.58%-1.95%) for dark skin (Tables S3 and S4 in Multimedia Appendix 1). Subgroup analyses found no statistically significant difference between light and medium skin pigmentation (estimate=0.113, SE 0.259; 95% CI –0.459 to 0.686), but there was a statistically significant difference between light and dark skin pigmentation (estimate=0.596, SE 0.240; 95% CI 0.069-1.123), such that pooled bias for those with darker skin was higher as compared to those with lighter skin (Table S2 in Multimedia Appendix 1).

**Table 4:** Pulse rate and pulse oximetry bias by skin pigmentation^a^.

Device type and skin pigment category		Studies (evaluations), n	Sample size (data pairs), n	Unit	Pooled mean bias (95% CI)	Pooled SD (SE)	95% LoA^b^	A_rms_^c^ (%)	Overall *I*^2^ (between- and within-study heterogeneity)
**Pulse oximetry**									
	Light	20 (44)	40,416 (131,008)	Percent	*0.70 (0.17 to 1.22)* ^d^	3.90 (1.36)	–6.94 to 8.34	3.96^e^	97.45% (46.44% and 51.01%)
	Medium	9 (15)	9,967 (32,095)	Percent	0.27 (–0.64 to 1.19)	4.71 (1.71)	–8.95 to 9.50	4.71^e^	95.31% (81.16% and 14.14%)
	Dark	19 (36)	9,301 (34,250)	Percent	*1.27 (0.58 to 1.95)*	3.96 (1.30)	–6.49 to 9.02	4.15^e^	98.46% (0.00% and 98.46%)
	Combined	23 (95)	59,684 (197,353)	Percent	*0.82 (0.29 to 1.35)*	3.50 (1.28)	–6.04 to 7.68	3.60^e^	98.55% (14.53% and 84.02%)
**Pulse rate**									
	Light	3 (9)	31 (43,116)	bpm^f^	–1.24 (–5.31 to 2.83)	7.54 (2.13)	–16.02^g^ to 13.54^g^	—^h^	10.99% (0.00% and 10.99%)
	Medium	3 (9)	129 (90,733)	bpm	–0.89 (–3.70 to 1.93)	9.05 (1.75)	–18.62^g^ to 16.84^g^	—	25.01% (0.00% and 25.01%)
	Dark	1 (6)	16 (6,922)	bpm	–0.57 (–9.44 to 8.29)	16.89 (1.31)	–33.69^g^ to 32·54^g^	—	13.70% (N/A^i^ and 13.70%)
	Combined	4 (24)	176 (140,771)	bpm	–0.29 (–3.87 to 3.29)	8.64 (1.67)	–17.23^g^ to 16.65^g^	—	30.66% (26.76% and 3.90%)

^a^ρ=0.9 was used in correlated and hierarchical effect models to pool both mean bias and SD.

^b^LoA: limits of agreement.

^c^A_rms_: accuracy root-mean-square

^d^Italicization denotes statistical significance.

^e^Exceeds U.S. Food and Drug Administration guidance for pulse oximetry.

^f^bpm: beats per minute.

^g^Exceeds American National Standards Institute standards for pulse rate.

^h^Not applicable.

^i^N/A: not available.

#### PR Results

**Figure 5:**
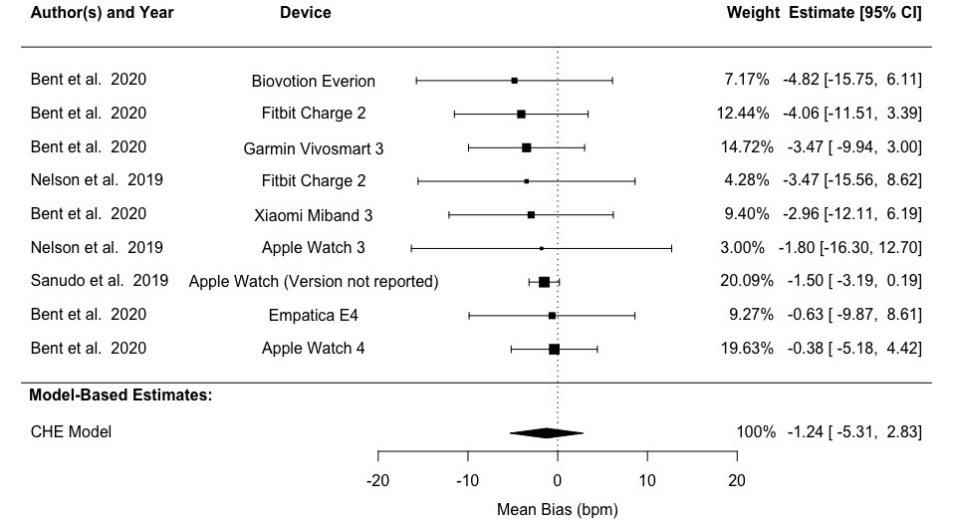
Forest plot showing pulse rate measurement bias in patients with light skin pigmentation. Squares denote study weight; center of squares denotes observed study effect size; vertical lines denote study CIs; and diamond denotes pooled effect. CHE: correlated and hierarchical effect.

**Figure 6:**
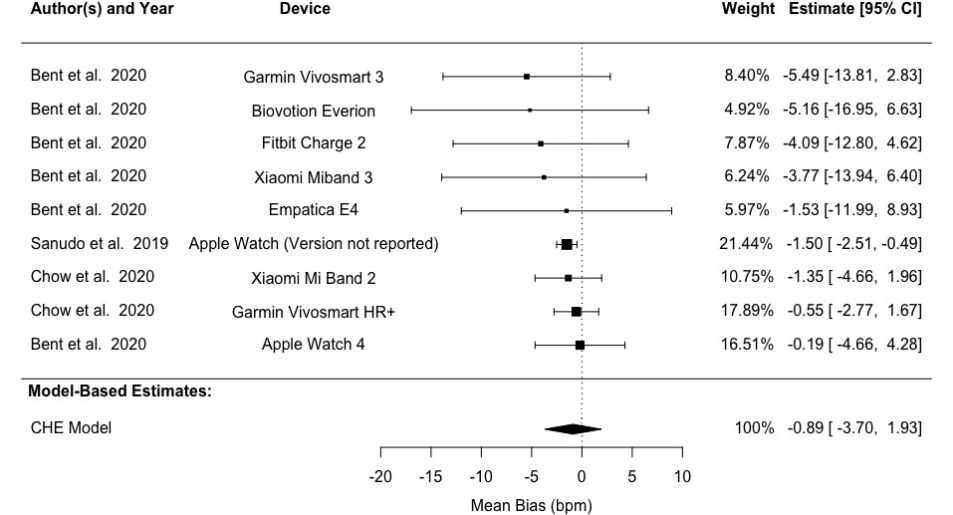
Forest plot showing pulse rate measurement bias in patients with medium skin pigmentation. Squares denote study weight; center of squares denotes observed study effect size; vertical lines denote study CIs; and diamond denotes pooled effect. CHE: correlated and hierarchical effect.

**Figure 7:**
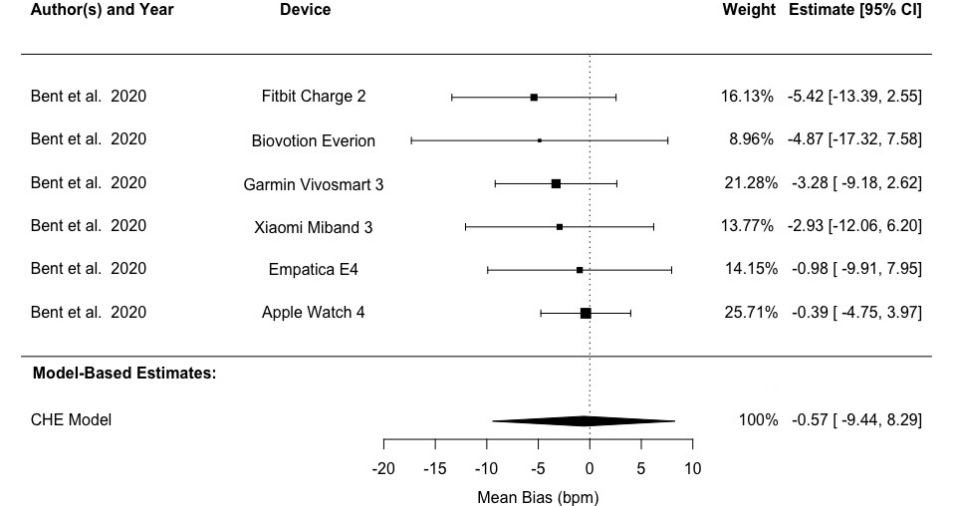
Forest plot showing pulse rate measurement bias in patients with dark skin pigmentation. Squares denote study weight; center of squares denotes observed study effect size; vertical lines denote study CIs; and diamond denotes pooled effect. CHE: correlated and hierarchical effect.

Analysis using the CHE model revealed LoA values of –17.23 to 16.65 bpm and a mean bias of –0.29 (95% CI –3.87 to 3.29) bpm across all studies. Heterogeneity analysis demonstrated that 26.76% of the variation in bias (*I*^2^_Level3_) stemmed from between-study differences, while 73.34% (*I*^2^_Level2_) originated from within-study variation. Our analysis revealed 95% LoA of –16.02 to 13.54 bpm, –18.62 to 16.84 bpm, and –33.69 to 32.54 bpm for light, medium, and dark skin pigmentation groups, respectively. Mean biases were –1.24 (95% CI –5.31 to 2.83) bpm, –0.89 (95% CI –3.70 to 1.93) bpm, and –0.57 (95% CI –9.44 to 8.29) bpm for the corresponding groups (Table 4 and Figures 5-7 [41,60-62]). Detailed results are provided in Tables S4 and S5 in Multimedia Appendix 1. Subgroup analyses found no statistically significant difference between light and medium or between light and dark skin pigmentation pooled bias (*P*≥.05; Table S2 in Multimedia Appendix 1).

#### Sensitivity Analyses

Sensitivity analyses were performed for pulse oximetry SpO_2_ by removing Crooks et al [48] as an outlier due to substantially large SE (Tables S6 and S7 in Multimedia Appendix 1). The pooled mean percent biases and 95% LoA were essentially unchanged, while the pooled A_rms_ across all skin pigmentation groups was 3.00%, 2.91%, and 3.40% for light, medium, and dark skin pigmentation, respectively. Sensitivity analyses using CHE models with a range of correlation coefficients (including small and moderate values) yielded similar conclusions to those obtained with a high correlation coefficient (ρ=0.9), except for the PR analysis in the light skin pigmentation group. This analysis revealed a statistically significant bias when a low correlation coefficient (ρ=0.3) was used. Details are provided in Table S6 in Multimedia Appendix 1.

## Discussion

### Principal Findings

The study revealed a paucity of studies properly assessing skin pigmentation, a tendency for uneven distribution across different skin pigmentation groups (overrepresentation of light skin for SpO_2_ and a single study with representation of dark skin for PR), and lack of consumer-device reporting despite growing use in clinical setting. Results suggest inaccurate SpO_2_ and PR measurements across all skin pigmentation groups as they breach FDA and ANSI/AAMI/IEC standards, respectively, with wearable accuracy varying considerably depending on the model, which may be due to date of model production or algorithm development. Pulse oximeters may also overestimate SpO_2_ significantly for light and dark skin pigmentation, but without clinically relevant bias. We did not find statistically significant or clinically relevant bias in wearable PR devices.

Despite not meeting FDA guidance across all groups, pulse oximeter SpO_2_ was inaccurate only across medium and dark skin pigmentation groups when compared to the more liberal international thresholds. Additionally, in the sensitivity analyses without the outlier study of Crooks et al [48], all pooled A_rms_ values dropped, resulting in inaccurate pulse oximeter SpO_2_ only for dark skin pigmentation and no group exceeding international thresholds.

The results showing pulse oximeters significantly overestimating SpO_2_ were expected for dark pigmentation and supported by findings on patient outcomes [11-15,17], but overestimated values for light pigmentation were unexpected. Two possible reasons come to mind. First, less melanin in lighter skin could distort the PPG signal [18]. Second, devices calibrated on individuals with medium skin pigmentation (note that 48% of the US population is categorized as Fitzpatrick Skin Tone Scale III [66]) may lead to inaccurate readings for both lighter and darker skin pigmentation, since both may be suboptimally represented during algorithm training and testing.

Lastly, it should be noted that both SpO_2_ and PR studies had lower between-study heterogeneity and higher within-study heterogeneity, indicating that the studies that were included in these analyses were largely consistent with one another and that most of the variation came from within studies potentially due to higher variability across devices used or participants enrolled within each study.

Overall, these findings suggest that when pulse oximetry devices are deployed in their setting of intended use (ie, uncontrolled settings, such real-world medical settings and home environments with diverse patient populations), the performance observed in analytical validation studies may not generalize.

### Strengths and Limitations

There were a few limitations, mostly from limitations inherent in prior studies, that should be noted. First, our skin pigmentation categorization approach has strengths but also limitations. It was an effort to overcome the reporting heterogeneity and the tendency in the published literature to conflate data collection on race, ethnicity, and skin tone. This is particularly problematic as skin tone is a physiological concept (determined by the melanin amount in the basal layer of the epidermis), while ethnicity and race are largely social constructs, with high underlying physiological heterogeneity [66]. To reduce heterogeneity, this meta-analysis reclassified race, ethnicity, and skin tone into a universal schema for skin pigmentation based on the system used by Shi et al [36]. This method, however, classifies most White people in the United States as light rather than medium skin pigmentation [66]. Second, there were only 4 prior PR studies that collected participant skin tone that also reported on device accuracy and only 1 that had participants with dark pigmentation. Third, our study used stated FDA and ANSI/AAMI/IEC standards as set thresholds to gauge device performance. It is possible that guidelines and thresholds cited in our study may change in the future, potentially limiting the applicability of the conclusions drawn in this paper. Fourth, our study chose to group papers using a variety of patient populations and testing methodologies with the goal of aggregating the largest pool of data possible on which to draw conclusions across multiple contexts. As described above, low between-study heterogeneity indicates that the studies used in this meta-analysis were largely consistent with one another. This reduces the likelihood of a moderating variable having a significant effect between studies. Instead, it suggests that most of the variation originated within studies, possibly due to higher variability across devices used or participants enrolled within each study. Despite this, we conducted subgroup analyses to examine the potential moderating variables of medical versus healthy populations for pulse oximeters (see Table S8 in Multimedia Appendix 1). We did not conduct these analyses for pulse rate because most studies did not specify whether subjects were healthy or part of medical populations. We did not examine the potential moderating effects of sensor type, transmittance versus reflectance sensors, wavelength of light used by sensors, and location of sensor placement given the core sensor technology studied was PPG across all included studies. The level of SpO_2_ was not examined given this study’s goal of gathering the largest sample upon which to study the effect of skin tone. Participant activity level was not evaluated as a moderator variable because many included studies did not delineate participant activity level explicitly. Future studies should evaluate the effect of these and other moderating variables on SpO_2_ and PR.

### Evidence Generation Guidelines for Future Analytical Validation Studies

The 2013 FDA guidance may be insufficient to ensure accuracy in pulse oximeters across all skin pigmentation and settings of intended use [19]. But there are now multiple FDA guidances for digital health tools requiring fit-for-purpose evidence as well as growing concern/guidance on clinical research diversity [4,30,31,67-73]. The FDA currently categorizes consumer devices as low-risk wellness products, exempting them from stringent regulatory oversight. However, as these devices are integrated into clinical decision-making and used as tools in clinical research [3,4], it becomes crucial to understand and communicate their advantages and limitations. Increased reliance on consumer devices increases the demand for accurate devices whose performance features and potential impact on health outcomes are known with transparency. To generate fit-for-purpose evidence applicable to diverse population, here, we propose 5 recommendations based on FDA guidance and literature (Textbox 1) [4,30,31,67-73]:

**Recommendations for future analytical validation studies.**

**Recommendation 1**

It is vitally important for medical pulse oximetry devices as well as nonregulated research and consumer devices to incorporate the V3 framework [74] for sensor verification and analytical validation of derived SpO_2_ and PR values for regulatory submission before these metrics can be responsibly deployed in medical, consumer, and research settings.

**Recommendation 2**

When possible, analytically validate devices in settings of intended use [74], rather than relying on controlled laboratory settings where digits may be warmed prior to testing, and confirm device accuracy in all subgroups (sex, race, skin pigmentation, healthy vs medical populations).

**Recommendation 3**

Use objective measures of skin pigmentation, rather than relying on race and ethnicity, as this will reduce heterogeneity in studies and allow for a more accurate understanding of how skin pigmentation impacts device performance.

**Recommendation 4**

Industry should set a priori maximum allowable difference thresholds using FDA and ANSI guidelines, properly power each subgroup, and require that 95% LoA fit within these standards for each subgroup and in the setting of intended use before receiving regulatory approval, production, and deployment.

**Recommendation 5**

Future studies should report device and firmware versions, as firmware updates may include changes in underlying algorithms influencing accuracy of metric generation, as described previously in the literature [4].

### Conclusions

PPG has been applied in clinical practice for decades, and its accuracy in patients with different skin pigmentation has long been in question. Whether this technology contributes to diagnostic biases and by how much is only more pressing for clinicians and patients with the advent of consumer wearable PPG sensors and the growing interest and incorporation of these devices into clinical practice and in clinical research. This systematic review and meta-analysis found that pulse oximeter SpO_2_ and wearable PR were inaccurate across all skin pigmentation groups as the resulting accuracy values breached FDA guidance and ANSI/AAMI/IEC standard thresholds, respectively, although pulse oximeter SpO_2_ was only found to be inaccurate for dark skin pigmentation in sensitivity analyses. In addition, despite not exceeding clinically relevant bias thresholds, pulse oximeters were found to significantly overestimate SpO_2_ for light and dark skin pigmentation. No systematic or clinically relevant bias was found in estimation of PR. The recommendations in this paper can help advise patients, study participants, care providers, device manufacturers, application developers, researchers, and legislators on best practices going forward.

## Appendix

Multimedia Appendix 1 Supplementary information.

Multimedia Appendix 2 PRISMA (Preferred Reporting Items for Systematic Reviews and Meta-Analyses) 2020 checklist.

## Abbreviations

AAMI: Association for the Advancement of Medical Instrumentation
ANSI: American National Standards Institute
A_rms_: accuracy root-mean-square
bpm: beats per minute
CHE: correlated and hierarchical effect
ECG: electrocardiography
FDA: Food and Drug Administration
IEC: International Electrotechnical Commission
LoA: limits of agreement
PPG: photoplethysmography
PR: pulse rate
PRIMSA: Preferred Reporting Items for Systematic Reviews and Meta-Analyses
RVE: robust variance estimation
SaO_2_: arterial blood gas
SpO_2_: blood oxygenation

## Data Availability

Open code and data are available on Open Science Framework [75] (see the Materials—Open Code and Data section in Multimedia Appendix 1).
