# Supplementary material for: Impact of Skin Pigmentation on Pulse Oximetry Blood Oxygenation and Wearable Pulse Rate Accuracy: Systematic Review and Meta-Analysis
Source: J Med Internet Res. 2024 Oct 10;26:e62769. doi: 10.2196/62769 (PMC11502980; doi:10.2196/62769)
Supplement: Multimedia Appendix 1 [file jmir_v26i1e62769_app1.docx]

**Multimedia Appendix 1**

**Impact of Skin Pigmentation on Pulse Oximetry Blood Oxygenation and Wearable Pulse Rate Accuracy: Systematic Review and Meta-Analysis**

Sanidhya Singh, B.S.E., Miles Bennett, M.S., Chen Chen, Ph.D, Sooyoon Shin, Ph.D., Hamid Ghanbari, M.D., Benjamin W. Nelson, Ph.D.

**Table of Contents**

**Tables 2**

Table S1. Schema for coding skin tone, race, and ethnicity to low, medium, and high skin pigmentation categories 2

Table S2. Subgroup analysis by skin pigmentation 3

Table S3. Pulse oximetry study bias by skin pigmentation 4

Table S4. Heterogeneity of pulse rate bias by skin pigmentation 10

Table S5. Pulse rate study bias by skin pigmentation 11

Table S6. Sensitivity analyses of pulse rate bias by skin pigmentation 13

Table S7. Pulse oximetry sensitivity analysis with Crooks et al. (2022) removed 14

Table S8. Subgroup analysis of pulse oximeter data comparing data from patients with medical conditions to that from healthy participants 15

Table S9. Shi et al. (2022)^1^ manuscripts not included 16

Table S10. Manuscripts exluded at full-text review stage 17

**Figures 25**

Figure S1. QUADAS-2 25

Figure S2. Funnel plots assessing publication bias in pulse oximetry studies, stratified by light, medium, and dark skin pigmentation 26

Figure S3. Funnel plots assessing publication bias in pulse rate studies, stratified by light, medium, and dark skin pigmentation 27

**Materials 28**

Search Report: PPG and Skin of Color 28

Data Extraction 32

Open Code and Data 32

Additional Details on Method for Statistical Analysis 33

**References 34**

# **Tables**

## **Table S1.** Schema for coding skin tone, race, and ethnicity to low, medium, and high skin pigmentation categories.

| **Skin pigmentation measurement methods** | **The number of classification categories as reported** | **Low (light) skin pigmentation** | **Medium skin pigmentation** | **High (dark) skin pigmentation** |
| --- | --- | --- | --- | --- |
| Fitzpatrick scale | Three categories | ‘Light (Type I to Type II)’, or ‘light (score of 1 or 2)’ | ‘Medium (Type III to  Type IV)’, or ‘medium (score of 3 or 4)’ | ‘Dark (Type V to Type VI)’, or ‘dark (score of 5 or 6)’ |
| Munsell color  system | Three categories | ‘Light’ | ‘Medium’ | ‘Dark’ |
| Using race and ethnicity to indicate skin pigmentation | Three categories | ‘Light (Caucasian)’ | ‘Intermediate (Hispanic, Indian, Filipino, Vietnamese)’ | ‘Dark (African American)’ |
| Objective quantification using a reflectance spectrophotometer | One category |  |  | ‘Dark pigmentation’ |

Note: Adapted from Shi et al. (2022) [36]

## **Table S2.** Subgroup analysis by skin pigmentation.

| **Device Type** | **Coefficient** | **Estimate** | **SE** | **p-value** | **95% CI (LL, UL)** |
| --- | --- | --- | --- | --- | --- |
| **Pulse Oximetry** | Intercept | 0.589 | 0.223 | **0.020** | **0.1068, 1.072** |
|  | Medium | 0.113 | 0.259 | 0.670 | -0.459, 0.686 |
|  | Dark | 0.596 | 0.240 | **0.030** | **0.069, 1.123** |
| **Pulse Rate** | Intercept | -0.163 | 0.970 | 0.883 | -4.584, 4.259 |
|  | Medium | -0.178 | 0.148 | 0.432 | -1.797, 1.441 |
|  | Dark | -0.215 | 0.067 | 0.150 | -0.747, 0.318 |

####

#### Note: Rho is set to 0.9; Reference is light; SE = standard error; LL = lower limit; UL = upper limit

## **Table S3.** Pulse oximetry study bias by skin pigmentation.

| **Study** | **Device** | **Sample** | **Data Pairs** | **Mean Bias** | **SE** | **Skin Pigmentation Group** |
| --- | --- | --- | --- | --- | --- | --- |
| Adler et al. 1998 | Nellcor D-25 | 145 | 145 | 2·50 | 0·38 | light |
| Andrist et al. 2022 | Not Specified | 878 | 7018 | 3·50 | 0·17 | light |
| Barker et al. 2023 | Masimo SET¬Æ pulse oximeters | 36 | 3982 | -0·05 | 0·23 | light |
| Bickler et al. 2005 | Nonin Onyx | 10 | 200 | -0·21 | 0·47 | light |
| Bickler et al. 2005 | Novametrix 513 | 10 | 200 | 1·15 | 0·60 | light |
| Bickler et al. 2005 | Nellcor D-25 | 10 | 220 | -0·27 | 0·68 | light |
| Burnett et al. 2022 | Not Specified- Hospital Standard Pulse Oximeter | 22089 | 70722 | 0·20 | 0·04 | light |
| Crooks et al. 2022 | Not Specified- Hospital Standard Pulse Oximeter | 3946 | 3946 | 3·20 | 13·24 | light |
| Ebmeier et al. 2018 | Philips (IntelliVue MP70) | 98 | 98 | -0·17 | 0·22 | light |
| Ebmeier et al. 2018 | Masimo (GE Marquette Rac-4A) | 142 | 142 | 1·07 | 0·16 | light |
| Fawzy et al. 2022 | Not Specified- Hospital Standard Pulse Oximeter | 460 | 460 | 0·75 | 0·09 | light |
| Feiner et al. 2007 | Masimo Radical (Clip) | 12 | 263 | 0·21 | 0·52 | light |
| Feiner et al. 2007 | Nellcor N-595 (clip) | 12 | 263 | 0·36 | 0·50 | light |
| Feiner et al. 2007 | Nonin 9700 (clip) | 12 | 263 | -0·95 | 0·45 | light |
| Feiner et al. 2007 | Masimo Radical (adhesive) | 12 | 263 | -0·64 | 0·68 | light |
| Feiner et al. 2007 | Nellcor N-595 & OxiMax (adhesive) | 12 | 263 | 1·04 | 0·47 | light |
| Feiner et al. 2007 | Nonin 9700 (adhesive) | 12 | 263 | 0·52 | 0·41 | light |
| Foglia et al. 2017 | Masimo Radical (Rainbow) | 21 | 21 | 0·20 | 0·83 | light |
| Foglia et al. 2017 | Nellcor OxiMax | 19 | 19 | 3·00 | 1·15 | light |
| Jubran et al. 1990 | Nellcor/Ohmeda Biox 3700 | 25 | 55 | 2·20 | 0·36 | light |
| McGovern et al. 1996 | IL 482 Co-oximeter | 8 | 8 | -1·7 | 1·03 | light |
| Munoz et al. 2008 | Minolta pulse oximeter | 846 | 846 | 0·47 | 0·08 | light |
| Pilcher et al. 2020 | 14 models | 242 | 242 | -1·2 | 0·11 | light |
| Ruppel et al. 2023 | Not Specified- Hospital Standard Pulse Oximeter | 573 | 573 | 0·88 | 0·13 | light |
| Sudat et al. 2023 | Not Specified- Hospital Standard Pulse Oximeter | 10514 | 35127 | 1·53 | 0·05 | light |
| Thrush et al. 1994 | Dinamap Plus 8700 | 22 | 88 | 0·00 | 0·65 | light |
| Thrush et al. 1994 | Dinamap Plus 8700 | 22 | 88 | 1·00 | 0·43 | light |
| Thrush et al. 1994 | Dinamap Plus 8700 | 22 | 88 | 1·00 | 0·43 | light |
| Thrush et al. 1994 | Dinamap Plus 8700 | 22 | 88 | 1·00 | 0·22 | light |
| Thrush et al. 1994 | Ohmeda Oxyshuttle | 22 | 88 | 2·00 | 0·65 | light |
| Thrush et al. 1994 | Ohmeda Oxyshuttle | 22 | 88 | 1·00 | 0·65 | light |
| Thrush et al. 1994 | Ohmeda Oxyshuttle | 22 | 88 | 0·00 | 0·43 | light |
| Thrush et al. 1994 | Ohmeda Oxyshuttle | 22 | 88 | 1·00 | 0·22 | light |
| Thrush et al. 1994 | MiniOx IV | 22 | 88 | 1·00 | 0·65 | light |
| Thrush et al. 1994 | MiniOx IV | 22 | 88 | 0·00 | 0·87 | light |
| Thrush et al. 1994 | MiniOx IV | 22 | 88 | 1·00 | 0·43 | light |
| Thrush et al. 1994 | MiniOx IV | 22 | 88 | 0·00 | 0·43 | light |
| Thrush et al. 1994 | Ohmeda 3700 | 22 | 88 | 1·00 | 1·08 | light |
| Thrush et al. 1994 | Ohmeda 3700 | 22 | 88 | 0·00 | 0·65 | light |
| Thrush et al. 1994 | Ohmeda 3700 | 22 | 88 | 2·00 | 0·43 | light |
| Thrush et al. 1994 | Ohmeda 3700 | 22 | 88 | 1·00 | 0·43 | light |
| Valbuena et al. 2022 | Not Specified- Hospital Standard Pulse Oximeter | 186 | 675 | 0·30 | 0·54 | light |
| Vesoulis et al. 2021 | nelcor MAX-N | 170 | 2342 | 0·72 | 0·68 | light |
| Wiles et al. 2022 | Nelcor GE B1x5 M/P | 135 | 4197 | -0·28 | 0·09 | light |
| Adler et al. 1998 | Nellcor D-25 | 105 | 105 | 2·80 | 0·51 | medium |
| Burnett et al. 2022 | Not Specified- Hospital Standard Pulse Oximeter | 8916 | 29344 | -0·41 | 0·08 | medium |
| Crooks et al. 2022 | Not Specified- Hospital Standard Pulse Oximeter | 246 | 246 | 5·10 | 14·74 | medium |
| Ebmeier et al. 2018 | Philips (IntelliVue MP70) | 89 | 89 | -0·45 | 0·22 | medium |
| Ebmeier et al. 2018 | Masimo (GE Marquette Rac-4A) | 48 | 48 | -0·11 | 0·26 | medium |
| Fawzy et al. 2022 | Not Specified- Hospital Standard Pulse Oximeter | 278 | 278 | 0·79 | 0·14 | medium |
| Feiner et al. 2007 | Masimo Radical (clip) | 7 | 154 | 0·99 | 0·85 | medium |
| Feiner et al. 2007 | Nellcor N-595 (clip) | 7 | 154 | 0·64 | 0·65 | medium |
| Feiner et al. 2007 | Nonin 9700 (clip) | 7 | 154 | 0·03 | 0·78 | medium |
| Feiner et al. 2007 | Masimo Radical (adhesive) | 7 | 154 | -0·34 | 0·80 | medium |
| Feiner et al. 2007 | Nellcor N-595 & OxiMax (adhesive) | 7 | 154 | 0·83 | 0·95 | medium |
| Feiner et al. 2007 | Nonin 9700 (adhesive) | 7 | 154 | 0·51 | 1·07 | medium |
| Pilcher et al. 2020 | 14 models | 157 | 157 | -1·10 | 0·13 | medium |
| Valbuena et al. 2022 | Not Specified- Hospital Standard Pulse Oximeter | 135 | 481 | 0·27 | 0·53 | medium |
| Wiles et al. 2022 | Not Specified | 34 | 1241 | 0·33 | 0·19 | medium |
| Abrams et al. 2002 | Nellcor N200 | 16 | 16 | 3·60 | 0·33 | dark |
| Adler et al. 1998 | Nellcor D-25 | 34 | 34 | 2·20 | 0·63 | dark |
| Andrist et al. 2022 | Not Specified | 183 | 2005 | 4·30 | 0·37 | dark |
| Barker et al. 2023 | Masimo SET¬Æ pulse oximeters | 39 | 3201 | -0·20 | 0·23 | dark |
| Bickler et al. 2005 | Nonin Onyx | 11 | 119 | 0·15 | 0·47 | dark |
| Bickler et al. 2005 | Novametrix 513 | 11 | 119 | 1·83 | 0·67 | dark |
| Bickler et al. 2005 | Nellcor 5-595 | 11 | 209 | 1·29 | 0·64 | dark |
| Bothma et al. 1996 | Simed S100e | 100 | 100 | 1·20 | 0·19 | dark |
| Bothma et al. 1996 | Nihon Koden | 100 | 100 | 0·85 | 0·25 | dark |
| Bothma et al. 1996 | Ohmeda 3740 (finger) | 100 | 100 | 0·55 | 0·20 | dark |
| Bothma et al. 1996 | Ohmeda 3740 (ear) | 100 | 100 | -1·00 | 0·24 | dark |
| Burnett et al. 2022 | Not Specified- Hospital Standard Pulse Oximeter | 5177 | 16011 | -0·60 | 0·13 | dark |
| Crooks et al. 2022 | Not Specified- Hospital Standard Pulse Oximeter | 151 | 151 | 5·40 | 15·99 | dark |
| Ebmeier et al. 2018 | IntelliVue MP70 | 8 | 8 | -2·24 | 0·61 | dark |
| Ebmeier et al. 2018 | GE Marquette Rac-4A | 4 | 4 | -1·10 | 0·20 | dark |
| Fawzy et al. 2022 | Not Specified- Hospital Standard Pulse Oximeter | 478 | 478 | 1·16 | 0·13 | dark |
| Feiner et al. 2007 | Masimo Radical (clip) | 17 | 372 | 2·14 | 0·73 | dark |
| Feiner et al. 2007 | Nellcor N-595 (clip) | 17 | 371 | 2·04 | 0·52 | dark |
| Feiner et al. 2007 | Nonin 9700 (clip) | 17 | 372 | -0·63 | 0·29 | dark |
| Feiner et al. 2007 | Masimo Radical (adhesive) | 17 | 372 | -0·24 | 0·70 | dark |
| Feiner et al. 2007 | Nellcor N-595 (Oximax, adhesive) | 17 | 359 | 2·13 | 0·60 | dark |
| Feiner et al. 2007 | Nonin 9700 (adhesive) | 17 | 371 | 1·26 | 0·52 | dark |
| Foglia et al. 2017 | Masimo Radical | 14 | 14 | 1·60 | 1·28 | dark |
| Foglia et al. 2017 | Nellcor Oximax | 14 | 14 | 5·40 | 1·36 | dark |
| Jubran et al. 1990 | Nellcor/Ohmeda Biox 3700 | 29 | 43 | 3·30 | 0·50 | dark |
| Ruppel et al. 2023 | Not Specified- Hospital Standard Pulse Oximeter | 201 | 201 | 2·61 | 0·22 | dark |
| Sudat et al. 2023 | Not Specified- Hospital Standard Pulse Oximeter | 2616 | 8626 | 2·45 | 0·14 | dark |
| Valbuena et al. 2022 | Not Specified- Hospital Standard Pulse Oximeter | 51 | 195 | 1·70 | 1·44 | dark |
| Vesoulis et al. 2021 | Nellcor MAX-N | 124 | 2044 | 1·73 | 0·84 | dark |
| Wiles et al. 2022 | Not Specified | 19 | 599 | 0·75 | 0·33 | dark |
| Zeballos et al. 1991 | Hewlett-Packard 47201A | 33 | 33 | -0·40 | 0·30 | dark |
| Zeballos et al. 1991 | Ohmeda Biox IIA | 33 | 33 | 2·10 | 0·35 | dark |
| Zeballos et al. 1991 | Hewlett-Packard 47201A | 11 | 11 | -0·80 | 0·51 | dark |
| Zeballos et al. 1991 | Ohmeda Biox IIA | 11 | 11 | 3·50 | 0·84 | dark |
| Zeballos et al. 1991 | Hewlett-Packard 47201A | 22 | 22 | -4·80 | 1·47 | dark |
| Zeballos et al. 1991 | Ohmeda Biox IIA | 22 | 22 | 9·80 | 1·47 | dark |

## **Table S4.** Heterogeneity of pulse rate bias by skin pigmentation.

| **Device Type** | **Skin Tone Category** | **Level** | **Percent of Total Variance** | **I^2^** | $\boldsymbol{\tau}$^2^ |
| --- | --- | --- | --- | --- | --- |
| **Pulse Rate** | Light | Level 1 | 89·00% | - | 9·62 |
|  |  | Level 2 | 11·00% | 11 | 1·19 |
|  |  | Level 3 | 0·00% | 0 | 0·00 |
|  | Medium | Level 1 | 74·99% | - | 3·24 |
|  |  | Level 2 | 25·01% | 25·01 | 1·08 |
|  |  | Level 3 | 0·00% | 0 | 0·00 |
|  | Dark* | Level 1 | 86·30% | - | 13·65 |
|  |  | Level 2 | 13·70% | 13·7 | 2·17 |
|  |  | Level 3 | - | - | - |
|  | Combined | Level 1 | 69·34% | - | 4·24 |
|  |  | Level 2 | 3·90% | 20·29 | 0·24 |
|  |  | Level 3 | 26·76% | 0·87 | 1·64 |
| **Pulse Oximetry** | Light | Level 1 | 2·55% | - | 0·03 |
|  |  | Level 2 | 51·01% | 51·01 | 0·61 |
|  |  | Level 3 | 46·44% | 46·44 | 0·55 |
|  | Medium | Level 1 | 4·69% | - | 0·05 |
|  |  | Level 2 | 14·14% | 14·14 | 0·16 |
|  |  | Level 3 | 81·16% | 81·16 | 0·94 |
|  | Dark | Level 1 | 1·53% | - | 0·08 |
|  |  | Level 2 | 98·46% | 98·62 | 5·48 |
|  |  | Level 3 | 0·00% | 0 | 0·00 |
|  | Combined | Level 1 | 1·45% | - | 0·04 |
|  |  | Level 2 | 84·02% | 84·02 | 2·37 |
|  |  | Level 3 | 14·53% | 14·53 | 0·41 |

Note: Rho = 0·9, * = Pulse Rate only had one study for dark skin tone and was a two-level model

## **Table S5.** Pulse rate study bias by skin pigmentation.

| **Study** | **Device** | **Sample** | **Data Pairs** | **Mean Bias** | **SE** | **Skin Pigmentation Group** |
| --- | --- | --- | --- | --- | --- | --- |
| Bent et al. 2020 | Empatica E4 | 15 | 41655 | -0·63 | 4·71 | light |
| Bent et al. 2020 | Fitbit Charge 2 | 15 | 3127 | -4·06 | 3·80 | light |
| Bent et al. 2020 | Biovotion Everion | 14 | 48367 | -4·82 | 5·57 | light |
| Bent et al. 2020 | Apple Watch 4 | 15 | 3841 | -0·38 | 2·45 | light |
| Bent et al. 2020 | Xiaomi Miband 3 | 15 | 6724 | -2·96 | 4·67 | light |
| Bent et al. 2020 | Garmin Vivosmart 3 | 15 | 11188 | -3·47 | 3·30 | light |
| Nelson et al. 2019 | Fitbit Charge 2 | 1 | 1446 | -3·47 | 6·17 | light |
| Nelson et al. 2019 | Apple Watch 3 | 1 | 394 | -180 | 7·40 | light |
| Sanudo et al. 2019 | Apple Watch (Version not reported) | 15 | 15 | -1·50 | 0·86 | light |
| Bent et al. 2020 | Apple Watch 4 | 19 | 4263 | -0·19 | 2·28 | medium |
| Bent et al. 2020 | Fitbit Charge 2 | 19 | 3763 | -4·09 | 4.44 | medium |
| Bent et al. 2020 | Garmin Vivosmart 3 | 19 | 12988 | -5·49 | 4·24 | medium |
| Bent et al. 2020 | Xiaomi Miband 3 | 19 | 6288 | -3·77 | 5·19 | medium |
| Bent et al. 2020 | Empatica E4 | 19 | 48843 | -1·53 | 5·34 | medium |
| Bent et al. 2020 | Biovotion Everion | 15 | 48312 | -5·16 | 6·01 | medium |
| Chow et al. 2020 | Garmin Vivosmart HR+ | 80 | 86440 | -0·55 | 1·13 | medium |
| Chow et al. 2020 | Xiaomi Mi Band 2 | 80 | 86440 | -1·35 | 1·69 | medium |
| Sanudo et al. 2019 | Apple Watch (Version not reported) | 30 | 30 | -1·50 | 0·51 | medium |
| Bent et al. 2020 | Xiaomi Miband 3 | 16 | 6922 | -2·93 | 4·66 | dark |
| Bent et al. 2020 | Biovotion Everion | 16 | 49194 | -4·87 | 6·35 | dark |
| Bent et al. 2020 | Garmin Vivosmart 3 | 18 | 12313 | -3·28 | 3·01 | dark |
| Bent et al. 2020 | Apple Watch 4 | 19 | 4631 | -0·39 | 2·22 | dark |
| Bent et al. 2020 | Fitbit Charge 2 | 19 | 3512 | -5·42 | 4·07 | dark |
| Bent et al. 2020 | Empatica E4 | 19 | 45744 | -0·98 | 4·56 | dark |

## **Table S6.** Sensitivity analyses of pulse rate bias by skin pigmentation.

| **⍴** | **Device Type** | **Skin Pigmentation Category** | **Unit** | **Pooled Mean Bias (95% CI)** | **Pooled SD (SE)** | **95% LoA** | **A_rms_ %** | **Overall *I*^2^ (between and within heterogeneity)** |
| --- | --- | --- | --- | --- | --- | --- | --- | --- |
| 0·3 | Pulse Oximetry | Light | percent | **0·70 (0·16 to 1·24)** | 3·90 (1·36) | -6·93 to 8·34 | 3·96^**^ | 97·52% (67·25% and 30·27%) |
|  |  | Medium | percent | 0·29 (-0·63 to 1·21) | 4·71 (1·71) | -9·93 to 9·51 | 4·71^**^ | 95·32% (94·51% and 0·81%) |
|  |  | Dark | percent | **1·27 (0·61 to 1·94)** | 3·96 (1·30) | -6·48 to- 9·03 | 4·15^**^ | 98·04 (0·00% and 98·04%) |
| 0·3 | Pulse Rate | Light | bpm | **-1·54 (-2·57 to -0·52)** | 7·54 (2·13) | -16·32^*^ to 13·23^*^ | – | 0·00% (0·00% and 0·00%) |
|  |  | Medium | bpm | -1·34 (-3·94 to 1·25) | 9·05 (1·75) | -19·07^*^ to 16·39^*^ | – | 0·00% (0·00% and 0·00%) |
|  |  | Dark | bpm | -1·43 (-16·49 to 13·6) | 16·89 (1·31) | -34·54^*^ to 31·68^*^ | – | 0·00% (NA and 0·00%) |
| 0·6 | Pulse Oximetry | Light | percent | **0·70 (0·17 to 1·24)** | 3·90 (1·36) | -6·94 to 8·34 | 3·96^**^ | 97·50% (58·83% and 38·67%) |
|  |  | Medium | percent | 0·29 (-0·64 to 1·21) | 4·71 (1·71) | -8·93 to 9·51 | 4·71^**^ | 95·35% (92·49% and 2·86%) |
|  |  | Dark | percent | **1·27 (0·59** to **1·95)** | 3·96 (1·30) | -6·49 to 9·03 | 4·15^**^ | 98·26% (0·00% and 98·26%) |
| 0·6 | Pulse Rate | Light | bpm | -1·43 (-3·34 to 0·47) | 7·54 (2·13) | -16·21^*^ to 13·34^*^ | – | 0·00% (0·00% and 0·00%) |
|  |  | Medium | bpm | -1·28 (-5·05 to 2·49) | 9·05 (1·75) | -19·01^*^ to 16·45^*^ | – | 0·00% (0·00% and 0·00%) |
|  |  | Dark | bpm | -0·50 (-20·72 to 19·72) | 16·89 (1·31) | -33·61^*^ to 32·61^*^ | – | 0·00% (NA and 0·00%) |

Note: ⍴ was used in CHE models to pool both mean bias and SD; Bold = statistically significant; * = exceeds ANSI Standards for Pulse Rate; ** = exceeds FDA Guidance for Pulse Oximetry. Arms = accuracy root mean square, LoA = limits of agreement, CI = confidence interval, SD = standard deviation, SE = standard error.

## **Table S7.** Pulse oximetry sensitivity analysis with Crooks et al. (2022) removed.

| **Skin Tone Category** | **Pooled Mean Bias (95% CI)** | **Pooled SD (SE)** | **95% LoA** | **A_rms_ %** | **Overall *I*^2^ (between and within heterogeneity)** |
| --- | --- | --- | --- | --- | --- |
| Light | **0·70 (0·17 to 1·22)** | 2·91 (1·15) | -5·02 to 6·41 | 3·00 | 97·51% (46·5% and 51·01%) |
| Medium | 0·27 (-0·65 to 1·19) | 2·90 (1·28) | -5·41 to 5·95 | 2·91 | 95·63% (81·45% and 14·18%) |
| Dark | **1·26 (0·58 to 1·94)** | 3·16 (1·17) | -4·93 to 7·46 | 3·40^*^ | 98·51% (0·00% and 98·51%) |
| Combined | **0·82 (0·29 to 1·35)** | 2·85 (1·13) | -4·77 to 6·41 | 2·97 | 98·59% (14·37% and 84·23%) |

Note: ⍴= 0.9 was used in CHE models to pool both mean bias and SD; Bold = statistically significant; * = exceeds FDA Guidance for Pulse Oximetry. Arms = accuracy root mean square, LoA = limits of agreement, CI = confidence interval, SD = standard deviation, SE = standard error.

## **Table S8.** Subgroup analysis of pulse oximeter data comparing data from patients with medical conditions to that from healthy participants.

| **Device Type** | **Coefficient** | **Estimate** | **SE** | **p-value** | **95% CI (LL, UL)** |
| --- | --- | --- | --- | --- | --- |
| **Pulse Oximetry** | Intercept | 0.73 | 0.12 | **0.01** | **0.35, 1.11** |
|  | Medical | 0.21 | 0.39 | 0.61 | -0.77, 1.18 |
|  | Mixed | -0.85 | 0.12 | **0.01** | **-1.24, -0.47** |

#### Note: Rho is set to 0.9; Healthy is the reference group and there was only one study that had mixed healthy and medical patients; SE = standard error; LL = lower limit; UL = upper limit

## **Table S9**. Shi et al. (2022) [36] manuscripts not included.

| **Author** | **Reason** |
| --- | --- |
| Avant 1997 | Could not confirm if bias was calculated as test - reference or reference - test |
| Hinkelbein 2006 | Examined nail polish and not skin tone |
| Hinkelbein 2007 | Examined nail polish and not skin tone |

## **Table S10.** Manuscripts excluded at full-text review stage.

| **Study** | **Reason For Exclusion** |
| --- | --- |
| Banerjee et al. 2020^2^ | Abstract |
| Bhoyar et al. 2010^3^ | Abstract |
| Blanchet et al. 2021^4^ | Abstract |
| Foglia et al. 2016^5^ | Abstract |
| Kapur et al. 2011^6^ | Abstract |
| Nickel et al. 2018^7^ | Abstract |
| Sathiyakumar et al. 2011^8^ | Abstract |
| Vybornova et al. 2021^9^ | Abstract |
| Cui et al. 1990^10^ | Benchtop testing, no human subjects |
| Fallow et al. 2013^11^ | Benchtop testing, no human subjects |
| Mark et al. 2021^12^ | Benchtop testing, no human subjects |
| Avant et al. 1997^13^ | Cannot confirm how mean bias was calculated |
| Arnold et al. 2019^14^ | Collected race, but did not report on it |
| Gillinov et al. 2017^15^ | Collected race, but did not report on it |
| Huynh et al. 2021^16^ | Collected race, but did not report on it |
| Reddy et al. 2018^17^ | Collected race, but did not report on it |
| Seshadri et al. 2020^18^ | Collected race, but did not report on it |
| Hochstadt et al. 2020^19^ | Collected skin tone but did not report data categorized by skin tone |
| Webster et al. 2021^20^ | Collected skin tone but did not report data categorized by skin tone |
| Giggins et al. 2022^21^ | Collected skin tone but did not report data categorized by skin tone |
| Støve and Hansen 2022^22^ | Combined Fitzpatrick Skin Scale Types 1-3 into one category |
| Blondel and Perdrix 2021^23^ | Could not obtain full text |
| Gutmann 2003^24^ | Could not obtain full text |
| Housset 1957^25^ | Could not obtain full text |
| Lee et al. 1992^26^ | Could not obtain full text |
| Yan et al. 2017^27^ | Device uses PPG and ECG |
| Scardulla et al. 2018^28^ | Device uses PPG and ECG |
| Mantri and Jokerst 2022^29^ | Device utilized audio and PPG |
| Jang et al. 2020^30^ | Did not comment on primary endpoints of interest |
| Nikseresht et al. 2021^31^ | Did not comment on primary endpoints of interest |
| Velichkovska et al. 2022^32^ | Did not comment on primary endpoints of interest |
| Mukherjee et al. 2018^33^ | Did not comment on primary endpoints of interest |
| Murphy and Omar 2018^34^ | Did not comment on primary endpoints of interest |
| Gibney et al. 2010^35^ | Did not comment on primary endpoints of interest |
| Huang et al. 2021^36^ | Did not comment on primary endpoints of interest |
| Nachman et al. 2022^37^ | Did not comment on primary endpoints of interest |
| Hussain 2009^38^ | Did not discuss PPG and/or skin tone |
| Kashimutt 2014^39^ | Did not discuss PPG and/or skin tone |
| Bonafide et al. 2020^40^ | Did not discuss PPG and/or skin tone |
| Lipchak and Chupov 2021^41^ | Did not discuss PPG and/or skin tone |
| Ojeda and Dela Cruz 2020^42^ | Did not discuss PPG and/or skin tone |
| Rahnama'i et al. 2006^43^ | Did not discuss PPG and/or skin tone |
| Rodriguez-Quinonez et al. 2012^44^ | Did not discuss PPG and/or skin tone |
| Sartor et al. 2021^45^ | Did not discuss PPG and/or skin tone |
| Vandeput et al. 1990^46^ | Did not discuss PPG and/or skin tone |
| Bangash et al. 2022^47^ | Does not provide required statistics |
| Henry et al. 2022^48^ | Does not provide required statistics |
| Hermand et al. 2019^49^ | Does not provide required statistics |
| Lee et al. 1993^50^ | Does not provide required statistics |
| Puranen et al. 2020^51^ | Does not provide required statistics |
| Ray et al. 2021^52^ | Does not provide required statistics |
| Ries et al. 1989^53^ | Does not provide required statistics |
| Shcherbina et al. 2017^54^ | Does not provide required statistics |
| Sjoding et al. 2020^55^ | Does not provide required statistics |
| Wiles et al. 2022^56^ | Does not provide required statistics |
| Wong et al. 2021^57^ | Does not provide required statistics |
| Brooks et al. 2020^58^ | Does not provide required statistics |
| Cecil et al. 1988^59^ | Does not provide required statistics |
| Escourrou et al. 1990^60^ | Does not provide required statistics |
| Espinosa et al. 2020^61^ | Does not provide required statistics |
| Etiwy et al. 2019^62^ | Does not provide required statistics |
| Gabrielczyk and Buist 1988^63^ | Does not provide required statistics |
| Harris et al. 2019^64^ | Does not provide required statistics |
| Harris et al. 2016^65^ | Does not provide required statistics |
| Harskamp et al. 2021^66^ | Does not provide required statistics |
| Horton et al. 2017^67^ | Does not provide required statistics |
| Menghini et al. 2019^68^ | Does not provide required statistics |
| Pasadyn et al. 2019^69^ | Does not provide required statistics |
| Ries et al. 1985^70^ | Does not provide required statistics |
| Ross et al. 2014^71^ | Does not provide required statistics |
| Schallom et al. 2018^72^ | Does not provide required statistics |
| Smyth et al. 1986^73^ | Does not provide required statistics |
| Spierer et al. 2015^74^ | Does not provide required statistics |
| Stewart and Rowbottom 1991^75^ | Does not provide required statistics |
| Valbuena et al. 2022^76^ | Does not provide required statistics |
| Wallen et al. 2016^77^ | Does not provide required statistics |
| Wang and Poh 1985^78^ | Does not provide required statistics |
| Pipek et al. 2021^79^ | Does not provide required statistics |
| Harrison et al. 2011^80^ | Duplicate record of another record this search |
| Harrison et al. 2011^80^ | Duplicate record of another record this search |
| Pipek et al. 2021^79^ | Duplicate record of another record this search |
| Ralston et al. 1991^81^ | Duplicate record of another record this search |
| Ralston et al. 1991^81^ | Duplicate record of another record this search |
| Wong et al. 2021^57^ | Duplicate record of another record this search |
| Sjoding et al. 2020^55^ | Duplicate record of another record this search |
| Adler et al. 1998^82^ | Duplicate record of another record this search |
| Avant et al. 1997^13^ | Duplicate record of another record this search |
| Bickler et al. 2005^83^ | Duplicate record of another record this search |
| Bothma et al. 1996^84^ | Duplicate record of another record this search |
| Brooks et al. 2020^58^ | Duplicate record of another record this search |
| Ebmeier et al. 2018^85^ | Duplicate record of another record this search |
| Escourrou et al. 1990^60^ | Duplicate record of another record this search |
| Feiner et al. 2007^86^ | Duplicate record of another record this search |
| Foglia et al. 2017^87^ | Duplicate record of another record this search |
| Gabrielczyk and Buist 1988^63^ | Duplicate record of another record this search |
| Harris et al. 2016^65^ | Duplicate record of another record this search |
| Harris et al. 2019^64^ | Duplicate record of another record this search |
| Harskamp et al. 2021^66^ | Duplicate record of another record this search |
| Hinkelbein et al. 2007^88^ | Duplicate record of another record this search |
| Hinkelbein et al. 2007^89^ | Duplicate record of another record this search |
| Jubran and Tobin 1990^90^ | Duplicate record of another record this search |
| Lee et al. 1993^50^ | Duplicate record of another record this search |
| Ries et al. 1985^70^ | Duplicate record of another record this search |
| Ries et al. 1989^53^ | Duplicate record of another record this search |
| Ross et al. 2014^71^ | Duplicate record of another record this search |
| Schallom et al. 2018^72^ | Duplicate record of another record this search |
| Smyth et al. 1986^73^ | Duplicate record of another record this search |
| Stewart and Rowbottom 1991^75^ | Duplicate record of another record this search |
| Valbuena et al. 2022^76^ | Duplicate record of another record this search |
| Vesoulis et al. 2022^91^ | Duplicate record of another record this search |
| Wiles et al. 2021^92^ | Duplicate record of another record this search |
| Zeballos and Weisman1991^93^ | Duplicate record of another record this search |
| Wong et al. 2022^94^ | Erratum |
| Sjoding et al. 2021^95^ | Erratum |
| Bent et al. 2021^96^ | Erratum |
| Whitehead-Clarke et al. 2021^97^ | Letter |
| Okunlola et al. 2022^98^ | Meta-analysis |
| Shi et al. 2022^99^ | Meta-analysis |
| Garrett et al. 2023^100^ | No skin tone, race, or ethnicity |
| Bai et al. 2018^101^ | No skin tone, race, or ethnicity |
| Bellenger et al. 2021^102^ | No skin tone, race, or ethnicity |
| Benedetti et al. 2021^103^ | No skin tone, race, or ethnicity |
| Berryhill et al. 2020^104^ | No skin tone, race, or ethnicity |
| Boudreaux et al. 2018^105^ | No skin tone, race, or ethnicity |
| Cadmus-Bertram et al. 2017^106^ | No skin tone, race, or ethnicity |
| Cao et al. 2022^107^ | No skin tone, race, or ethnicity |
| de Zambotti et al. 2016^108^ | No skin tone, race, or ethnicity |
| Dooley et al. 2017^109^ | No skin tone, race, or ethnicity |
| Gorny et al. 2017^110^ | No skin tone, race, or ethnicity |
| Held et al. 2022^111^ | No skin tone, race, or ethnicity |
| Hettiarachchi et al. 2019^112^ | No skin tone, race, or ethnicity |
| Kinnunen et al. 2020^113^ | No skin tone, race, or ethnicity |
| Kroll et al. 2016^114^ | No skin tone, race, or ethnicity |
| Mendelson and Ochs 1988^115^ | No skin tone, race, or ethnicity |
| Miller et al. 2022^116^ | No skin tone, race, or ethnicity |
| Muggeridge et al. 2021^117^ | No skin tone, race, or ethnicity |
| Nuuttila et al. 2021^118^ | No skin tone, race, or ethnicity |
| Renevey et al. 2018^119^ | No skin tone, race, or ethnicity |
| Sarhaddi et al. 2022^120^ | No skin tone, race, or ethnicity |
| Schubert et al. 2018^121^ | No skin tone, race, or ethnicity |
| Schuurmans et al. 2020^122^ | No skin tone, race, or ethnicity |
| Sen-Gupta et al. 2019^123^ | No skin tone, race, or ethnicity |
| Stucky et al. 2021^124^ | No skin tone, race, or ethnicity |
| Wang et al. 2017^125^ | No skin tone, race, or ethnicity |
| List et al. 1991^126^ | Non-English text |
| Dyer 2020^127^ | Not a primary study (commentary on another study) |
| Ferrari et al. 2022^128^ | Not a primary study (commentary on another study) |
| Holder and Wong 2022^129^ | Not a primary study (commentary on another study) |
| Norton 2022^130^ | Not a primary study (commentary on another study) |
| Papaioannou et al. 2017^131^ | Not a primary study (commentary on another study) |
| Philip et al. 2021^132^ | Not a primary study (commentary on another study) |
| Ploen et al. 2016^133^ | Oral presentation |
| Todd et al. 2021^134^ | Oral presentation |
| Mukherjee et al. 2020^135^ | Prototype device |
| Harrison et al. 2011^136^ | Published in textbook, not peer reviewed |
| Królak and 2020^137^ | Published in textbook, not peer reviewed |
| Sjoding et al. 2021^138^ | Reply |
| Tobin and Jubran 2022^139^ | Reply |
| Colvonen et al. 2021^140^ | Response |
| Agache and Dupond 1994^141^ | Review Paper |
| AlDallal 2019^142^ | Review paper |
| Fine et al. 2021^143^ | Review Paper |
| Hay 1987^144^ | Review Paper |
| Hunasikatti 2021^145^ | Review Paper |
| Kamal et al. 1989^146^ | Review paper |
| Knight et al. 2022^147^ | Review paper |
| Ralston et al. 1991^81^ | Review paper |
| Ray et al. 2021^148^ | Review paper |
| Taylor and Whitwam 1986^149^ | Review paper |
| Tobin and Jubran 2022^150^ | Review Paper |
| Ajmal et al. 2021^151^ | Simulation, no human subjects |
| Flynn et al. 2021^152^ | Simulation, no human subjects |
| Chatterjee and Kyriacou 2019^153^ | Simulation, no human subjects |
| Fine et al. 2020^154^ | Simulation, no human subjects |
| Hinkelbein et al. 2007^89^ | Studied the effect of nail polish and not skin tone |
| Hinkelbein et al. 2007^88^ | Studied the effect of nail polish and not skin tone |
| Emery 1987^155^ | Study noted significant difficulty in classifying subject skin tone |
| Baek et al. 2018^156^ | Studying pulse oximetry but reference is not blood gas |
| Mendelson et al. 1987^157^ | Studying pulse oximetry but reference is not blood gas |
| Olmo Arroyo et al. 2017^158^ | Studying pulse oximetry but reference is not blood gas |
| Bickler et al. 2013^159^ | Studying pulse oximetry but reference is not blood gas |
| Cahan et al. 1990^160^ | Studying pulse oximetry but reference is not blood gas |
| Smith and Hofmeyr 2019^161^ | Studying pulse oximetry but reference is not blood gas |
| Stell et al. 2022^162^ | Studying pulse oximetry but reference is not blood gas |
| Witting and Scharf 2008^163^ | Studying pulse oximetry but reference is not blood gas |
| Schröder et al. 2023^164^ | Studying pulse oximetry but reference is not blood gas |
| Ahmed et al. 2018^165^ | Studying pulse rate but reference is not ECG |
| Preejith et al. 2016^166^ | Studying pulse rate but reference is not ECG |
| Spaccarotella et al. 2022^167^ | Studying pulse rate but reference is not ECG |
| Powell et al. 2013^168^ | Survey data, not directly studying PPG |

# **Figures**

## **Figure S1**. QUADAS-2.

Note: D1 = Risk of Bias: Patient Selection, D2 = Risk of Bias: Index Test, D3 = Risk of Bias: Reference Standard, D4 = Risk of Bias: Flow and Timing, D5 = Applicability Concerns: Patient Selection, D6: Applicability Concerns: Index Test, D7 = Applicability Concerns: Reference Standard; Green = low bias; Red = high bias; Yellow = unclear bias.


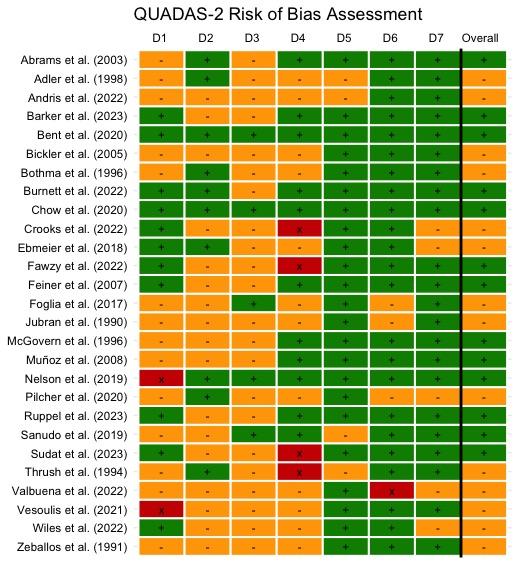


## **Figure S2.** Funnel plots assessing publication bias in pulse oximetry studies, stratified by light, medium, and dark skin pigmentation.

These plot individual study effect estimates against their respective precision, represented by the standard error. On these plots, the vertical axis shows the effect size, while the horizontal axis shows the standard error. When there is no bias, the plot looks like an inverted funnel; larger, more precise studies cluster towards the top, closer to the true effect size, and smaller, less precise studies spread towards the bottom, indicating a wider spread in effect estimates. Asymmetry in the funnel plot can hint at potential publication bias, but it might also indicate other biases or study heterogeneity.

| A.  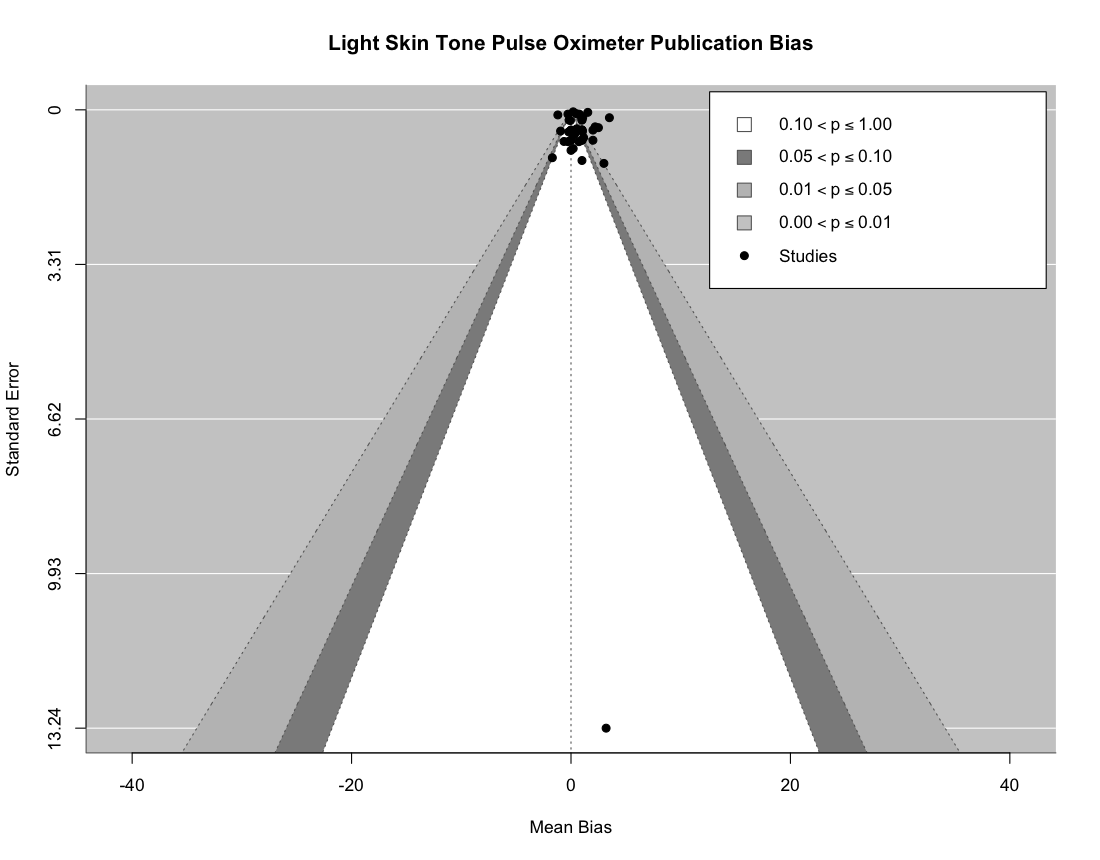 | B.  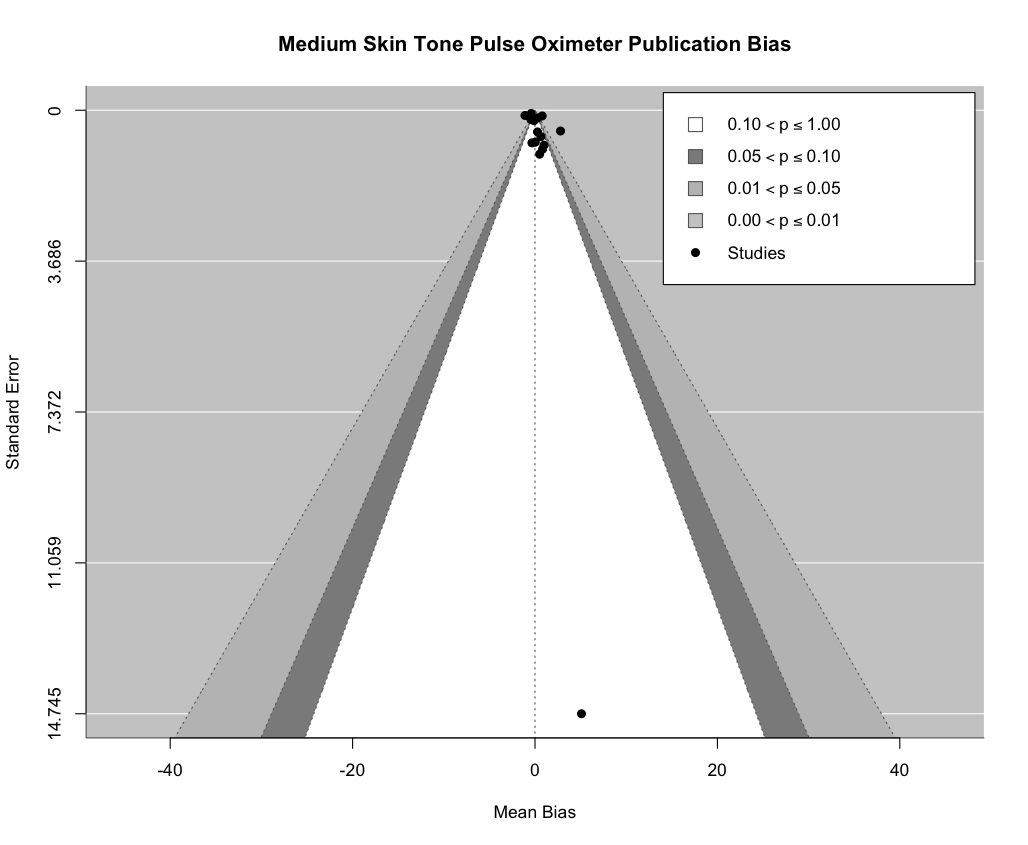 |
| --- | --- |
| C. 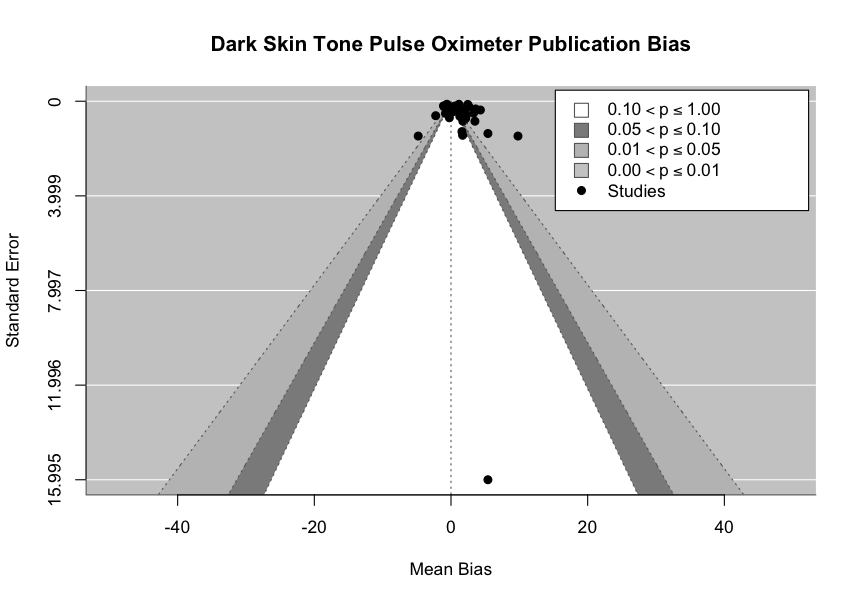 | |

####

## **Figure S3.** Funnel plots assessing publication bias in pulse rate studies, stratified by light, medium, and dark skin pigmentation.

These plot individual study effect estimates against their respective precision, represented by the standard error. On these plots, the vertical axis shows the effect size, while the horizontal axis shows the standard error. When there is no bias, the plot looks like an inverted funnel; larger, more precise studies cluster towards the top, closer to the true effect size, and smaller, less precise studies spread towards the bottom, indicating a wider spread in effect estimates. Asymmetry in the funnel plot can hint at potential publication bias, but it might also indicate other biases or study heterogeneity.

| A.  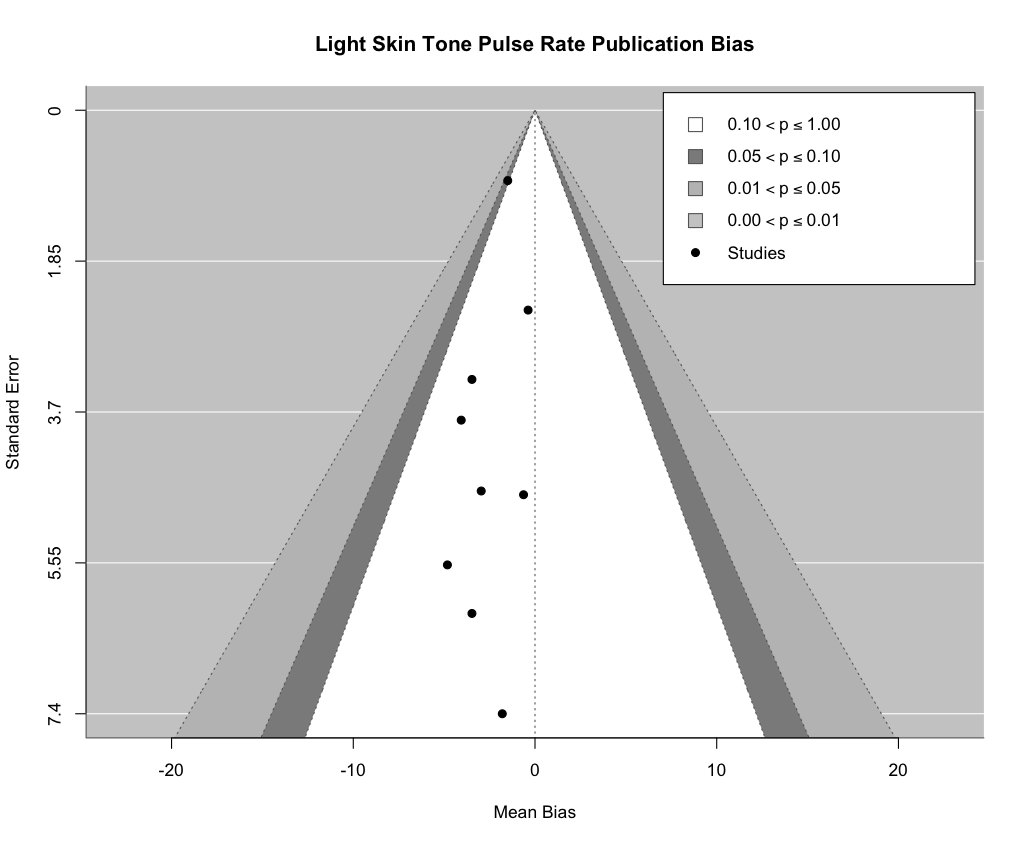 | B.  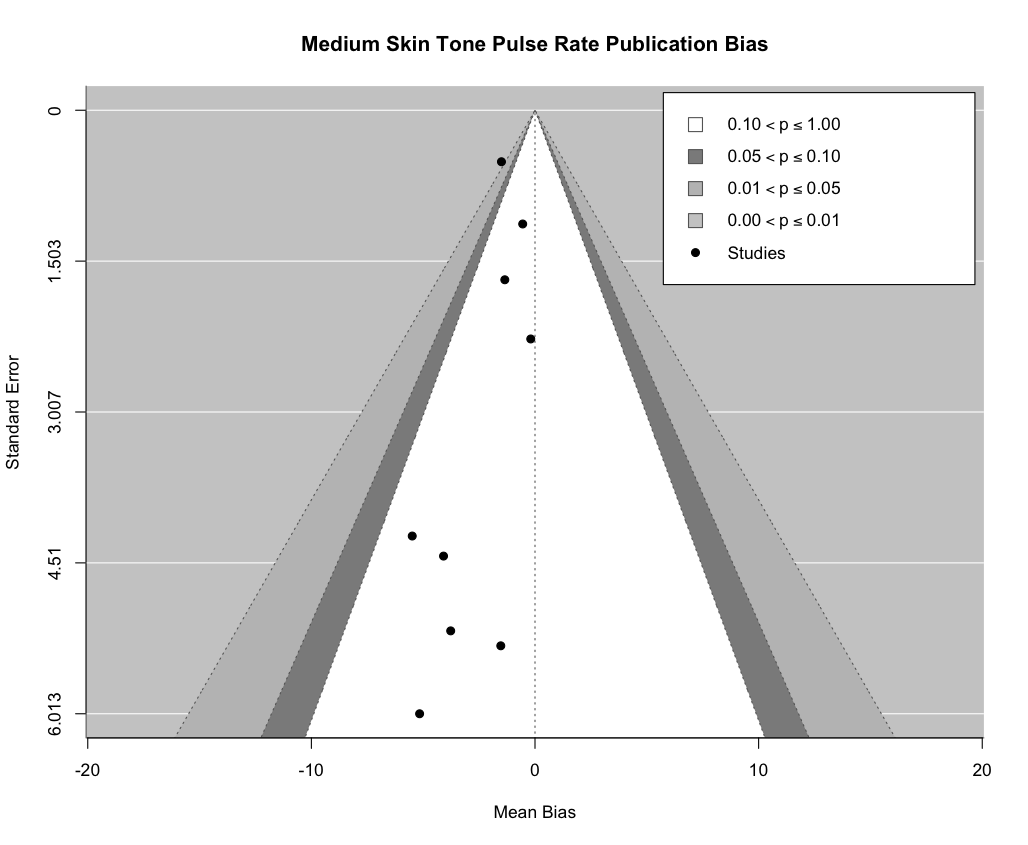 |
| --- | --- |
| C.  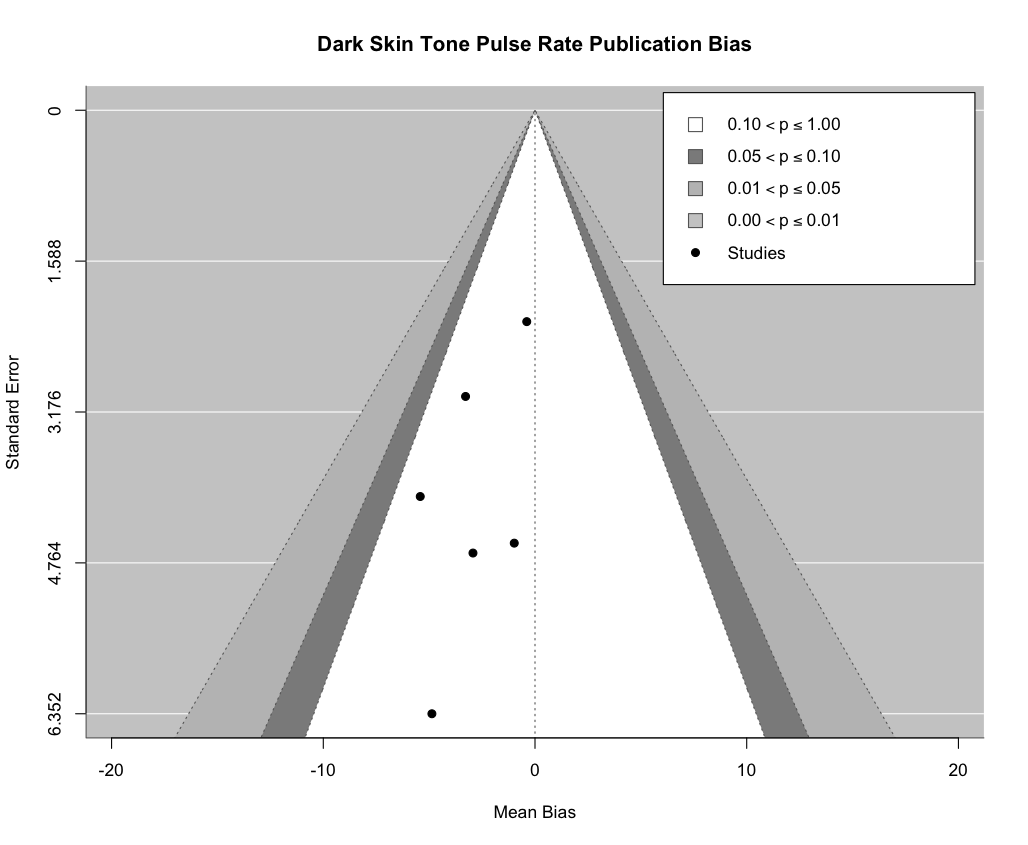 | |

# **Materials**

## **Search Report: PPG and Skin of Color**

Whitney A Townsend, MLIS

whitneyt@umich.edu

All searches run from inception of the database to 04/15/2022 unless otherwise noted

All searches run without limits unless otherwise noted

Original PubMed search strategy translated and adapted to other databases using http://sr-accelerator.com/#/polyglot and the searcher's discretion.

**Please cite:**

*SR Accelerator Polyglot search translation tool*^169^***.***

**Databases**

- PubMed (PubMed.gov)
- Embase.com (including Embase Classic)
- Scopus.com
- Web of Science Core Collection (SCI-EXPANDED, SSCI, A&HCI, CPCI-S, CPCI-SSH, BKCI-S, BKCI-SSH, ESCI, CCR-EXPANDED)
- CINAHL Complete (Ebsco)
- ClinicalTrials.gov

**Total results before deduplication: 8582**

**Total results after initial DistillerSR deduplication: 5082 (3500 duplicates removed)**

**SEARCH NOTES:**

Antiquated, non-standard, and potentially offensive terms for racial and ethnic groups were included in search strategies in order to conduct a sensitive, comprehensive search for relevant studies. Language has changed over time and varies geographically and by community, so a thorough search must include the terms that have been used in past and present literature. The authors recognize and acknowledge the inappropriate and harmful nature of these terms.

**PubMed - 1548 on 4/15/22**

(("Race Factors"[MeSH Terms] OR "Skin Pigmentation"[MeSH Terms] OR Racial Groups [MeSH Terms] OR "Indigenous Peoples"[MeSH Terms] OR "Arabs"[MeSH Terms] OR Jews[mesh] OR Roma[mesh] OR "Ethnicity"[MeSH Terms] OR

pigment*[tiab] OR racial[tiab] OR race[tiab] OR biracial[tiab] OR bi-racial[tiab] OR multiracial[tiab] OR multi-racial[tiab] OR "multiple race"[tiab] OR "multiple races"[tiab] OR racism[tiab] OR racist[tiab] OR African*[tiab] OR Black[tiab] OR Blacks[tiab] OR negro*[tiab] OR Native American*[tiab] OR Indian*[tiab] OR Indigenous[tiab] OR First Nation People*[tiab] OR "Native People"[tiab] OR Inuit*[tiab] OR Arab[tiab] OR Arabs[tiab] OR Arabic[tiab] OR "Middle East*"[tiab] OR Asian*[tiab] OR Asiatic*[tiab] OR Hispanic*[tiab] OR Latin*[tiab]

OR Hawaiian*[tiab] OR Pacific Islander*[tiab] OR Aborigin*[tiab] OR "people of color"[tiab] OR "people of colour"[tiab] OR BIPOC[tiab] OR "skin of color"[tiab] OR "skin of colour"[tiab] OR "brown skin"[tiab] OR "black skin"[tiab] OR "dark skin"[tiab] OR "dark skinned"[tiab] OR "darker skin"[tiab] OR melanin[tiab] OR ethnic*[tiab] OR skin tone*[tiab] OR skin color*[tiab] OR skin colour*[tiab] OR "skin phototypes"[tiab] OR hyperpigmentation[tiab] OR hypopigmentation[tiab] OR dyschromia[tiab]) AND (Photoplethysmography[MeSH Terms] OR PPG[tiab] OR

Photoplethysmogra*[tiab] OR Plethysmogra*[tiab] OR Optometric*[tiab] OR "Apple Watch"[tiab] OR Alivecor[tiab] OR Kardiamobile[tiab] OR Pulse oximeter*[tiab] OR pulse-ox*[tiab] OR Cuffless[tiab] OR "Empatica E4"[tiab] OR "Finger probe*"[tiab] OR Fitbit[tiab] OR "Smart watch*"[tiab] OR smartwatch*[tiab] OR Xiaomi[tiab] OR Biovotion[tiab] OR "Basis peak"[tiab] OR "Polar M600"[tiab] OR Misfit[tiab] OR Withings[tiab] OR Mio[tiab] OR Samsung[tiab] OR PulseON[tiab] OR TomTom[tiab] OR Empatica[tiab] OR Optoelectronic[tiab] OR "reflection index*"[tiab] OR "stiffness index*"[tiab])) NOT (animals[mesh] NOT humans[mesh])

**Embase - 2652 on 4/15/22**

((Race/exp OR "Skin Pigmentation"/exp OR "Ancestry Group"/exp OR "Ethnic Group"/exp OR Jew/exp OR Ethnicity/exp OR Racism/exp OR pigment*:ti,ab OR racial:ti,ab OR race:ti,ab OR biracial:ti,ab OR bi-racial:ti,ab OR multiracial:ti,ab OR multi-racial:ti,ab OR "multiple race":ti,ab OR "multiple races":ti,ab OR racism:ti,ab OR racist:ti,ab OR African*:ti,ab OR Black:ti,ab OR Blacks:ti,ab OR negro*:ti,ab OR "Native American*":ti,ab OR Indian*:ti,ab OR Indigenous:ti,ab OR "First Nation People*":ti,ab OR "Native People":ti,ab OR Inuit*:ti,ab OR Arab:ti,ab OR Arabs:ti,ab OR Arabic:ti,ab OR "Middle East*":ti,ab OR Asian*:ti,ab OR Asiatic*:ti,ab OR Hispanic*:ti,ab OR Latin*:ti,ab OR Hawaiian*:ti,ab OR "Pacific Islander*":ti,ab OR Aborigin*:ti,ab OR "people of color":ti,ab OR "people of colour":ti,ab OR BIPOC:ti,ab OR "skin of color":ti,ab OR "skin of colour":ti,ab OR "brown skin":ti,ab OR "black skin":ti,ab OR "dark skin":ti,ab OR "dark skinned":ti,ab OR "darker skin":ti,ab OR melanin:ti,ab OR ethnic*:ti,ab OR "skin tone*":ti,ab OR "skin color*":ti,ab OR "skin colour*":ti,ab OR "skin phototypes":ti,ab OR hyperpigmentation:ti,ab OR hypopigmentation:ti,ab OR dyschromia:ti,ab) AND ('photoelectric plethysmography'/exp OR PPG:ti,ab OR Photoplethysmogra*:ti,ab OR Plethysmogra*:ti,ab OR Optometric*:ti,ab OR "Apple Watch":ti,ab OR Alivecor:ti,ab OR Kardiamobile:ti,ab OR "Pulse oximeter*":ti,ab OR pulse-ox*:ti,ab OR Cuffless:ti,ab OR "Empatica E4":ti,ab OR "Finger probe*":ti,ab OR Fitbit:ti,ab OR "Smart watch*":ti,ab OR smartwatch*:ti,ab OR Xiaomi:ti,ab OR Biovotion:ti,ab OR "Basis peak":ti,ab OR "Polar M600":ti,ab OR Misfit:ti,ab OR Withings:ti,ab OR Mio:ti,ab OR Samsung:ti,ab OR PulseON:ti,ab OR TomTom:ti,ab OR Empatica:ti,ab OR Optoelectronic:ti,ab OR "reflection index*":ti,ab OR "stiffness index*":ti,ab)) NOT ([animals]/lim NOT [humans]/lim)

**Scopus - 2654 on 4/15/22**

( TITLE-ABS-KEY ( pigment* ) OR TITLE-ABS-KEY ( racial ) OR TITLE-ABS-KEY ( race ) OR TITLE-ABS-KEY ( biracial ) OR TITLE-ABS-KEY ( bi-racial ) OR TITLE-ABS-KEY ( multiracial ) OR TITLE-ABS-KEY ( multi-racial ) OR TITLE-ABS-KEY ( {multiple race} ) OR TITLE-ABS-KEY ( {multiple races} ) OR TITLE-ABS-KEY ( racism ) OR TITLE-ABS-KEY ( racist ) OR TITLE-ABS-KEY ( african* ) OR TITLE-ABS-KEY ( black W/3 skin ) OR TITLE-ABS-KEY ( negro* ) OR TITLE-ABS-KEY ( "Native American*" ) OR TITLE-ABS-KEY ( indian* ) OR TITLE-ABS-KEY ( indigenous ) OR TITLE-ABS-KEY ( "First Nation People*" ) OR TITLE-ABS-KEY ( {Native People} ) OR TITLE-ABS-KEY ( inuit* ) OR TITLE-ABS-KEY ( arab ) OR TITLE-ABS-KEY ( arabs ) OR TITLE-ABS-KEY ( arabic ) OR TITLE-ABS-KEY ( "Middle East*" ) OR TITLE-ABS-KEY ( asian* ) OR TITLE-ABS-KEY ( asiatic* ) OR TITLE-ABS-KEY ( hispanic* ) OR TITLE-ABS-KEY ( latin* ) OR TITLE-ABS-KEY ( hawaiian* ) OR TITLE-ABS-KEY ( "Pacific Islander*" ) OR TITLE-ABS-KEY ( aborigin* ) OR TITLE-ABS-KEY ( {people of color} ) OR TITLE-ABS-KEY ( {people of colour} ) OR TITLE-ABS-KEY ( bipoc ) OR TITLE-ABS-KEY ( {skin of color} ) OR TITLE-ABS-KEY ( {skin of colour} ) OR TITLE-ABS-KEY ( {brown skin} ) OR TITLE-ABS-KEY ( {black skin} ) OR TITLE-ABS-KEY ( {dark skin} ) OR TITLE-ABS-KEY ( {dark skinned} ) OR TITLE-ABS-KEY ( {darker skin} ) OR TITLE-ABS-KEY ( melanin ) OR TITLE-ABS-KEY ( ethnic* ) OR TITLE-ABS-KEY ( {skin tone} ) OR TITLE-ABS-KEY ( {skin tones} ) OR TITLE-ABS-KEY ( {skin color} ) OR TITLE-ABS-KEY ( {skin colors} ) OR TITLE-ABS-KEY ( {skin colour} ) OR TITLE-ABS-KEY ( {skin colours} ) OR TITLE-ABS-KEY ( {skin phototypes} ) OR TITLE-ABS-KEY ( hyperpigmentation ) OR TITLE-ABS-KEY ( hypopigmentation ) OR TITLE-ABS-KEY ( dyschromia ) )

AND

( TITLE-ABS-KEY ( ppg ) OR TITLE-ABS-KEY ( photoplethysmogra* ) OR TITLE-ABS-KEY ( plethysmogra* ) OR TITLE-ABS-KEY ( optometric* ) OR TITLE-ABS-KEY ( {Apple Watch} ) OR TITLE-ABS-KEY ( alivecor ) OR TITLE-ABS-KEY ( kardiamobile ) OR TITLE-ABS-KEY ( "Pulse oximeter*" ) OR TITLE-ABS-KEY ( pulse-ox* ) OR TITLE-ABS-KEY ( cuffless ) OR TITLE-ABS-KEY ( "Empatica E4" ) OR TITLE-ABS-KEY ( "Finger probe*" ) OR TITLE-ABS-KEY ( fitbit ) OR TITLE-ABS-KEY ( "Smart watch*" ) OR TITLE-ABS-KEY ( smartwatch* ) OR TITLE-ABS-KEY ( xiaomi ) OR TITLE-ABS-KEY ( biovotion ) OR TITLE-ABS-KEY ( "Basis peak" ) OR TITLE-ABS-KEY ( "Polar M600" ) OR TITLE-ABS-KEY ( withings ) OR TITLE-ABS-KEY ( samsung ) OR TITLE-ABS-KEY ( pulseon ) OR TITLE-ABS-KEY ( tomtom ) OR TITLE-ABS-KEY ( empatica ) OR TITLE-ABS-KEY ( optoelectronic ) OR TITLE-ABS-KEY ( "reflection index" ) OR TITLE-ABS-KEY ( "stiffness index" ) )

**Web of Science Core Collection - 1543 on 4/15/22**

((TS="Race Factors" OR TS="Skin Pigmentation" OR TS="Racial Groups" OR TS="Indigenous Peoples" OR TS=Arabs OR TS=Jews OR TS=Roma OR TS=Ethnicity OR (TI=pigment* OR AB=pigment*) OR (TI=racial OR AB=racial) OR (TI=race OR AB=race) OR (TI=biracial OR AB=biracial) OR (TI=bi-racial OR AB=bi-racial) OR (TI=multiracial OR AB=multiracial) OR (TI=multi-racial OR AB=multi-racial) OR (TI="multiple race" OR AB="multiple race") OR (TI="multiple races" OR AB="multiple races") OR (TI=racism OR AB=racism) OR (TI=racist OR AB=racist) OR (TI=African* OR AB=African*) OR ((TI=Black OR AB=Black) AND (TI=skin OR AB=skin)) OR (TI=negro* OR AB=negro*) OR (TI="Native American*" OR AB="Native American*") OR (TI=Indian* OR AB=Indian*) OR (TI=Indigenous OR AB=Indigenous) OR (TI="First Nation People*" OR AB="First Nation People*") OR (TI="Native People" OR AB="Native People") OR (TI=Inuit* OR AB=Inuit*) OR (TI=Arab OR AB=Arab) OR (TI=Arabs OR AB=Arabs) OR (TI=Arabic OR AB=Arabic) OR (TI="Middle East*" OR AB="Middle East*") OR (TI=Asian* OR AB=Asian*) OR (TI=Asiatic* OR AB=Asiatic*) OR (TI=Hispanic* OR AB=Hispanic*) OR (TI=Latin* OR AB=Latin*) OR (TI=Hawaiian* OR AB=Hawaiian*) OR (TI="Pacific Islander*" OR AB="Pacific Islander*") OR (TI=Aborigin* OR AB=Aborigin*) OR (TI="people of color" OR AB="people of color") OR (TI="people of colour" OR AB="people of colour") OR (TI=BIPOC OR AB=BIPOC) OR (TI="skin of color" OR AB="skin of color") OR (TI="skin of colour" OR AB="skin of colour") OR (TI="brown skin" OR AB="brown skin") OR (TI="black skin" OR AB="black skin") OR (TI="dark skin" OR AB="dark skin") OR (TI="dark skinned" OR AB="dark skinned") OR (TI="darker skin" OR AB="darker skin") OR (TI=melanin OR AB=melanin) OR (TI=ethnic* OR AB=ethnic*) OR (TI="skin tone*" OR AB="skin tone*") OR (TI="skin color*" OR AB="skin color*") OR (TI="skin colour*" OR AB="skin colour*") OR (TI="skin phototypes" OR AB="skin phototypes") OR (TI=hyperpigmentation OR AB=hyperpigmentation) OR (TI=hypopigmentation OR AB=hypopigmentation) OR (TI=dyschromia OR AB=dyschromia)) AND (ALL=Photoplethysmography OR (TI=PPG OR AB=PPG) OR (TI=Photoplethysmogra* OR AB=Photoplethysmogra*) OR (TI=Plethysmogra* OR AB=Plethysmogra*) OR (TI=Optometric* OR AB=Optometric*) OR (TI="Apple Watch" OR AB="Apple Watch") OR (TI=Alivecor OR AB=Alivecor) OR (TI=Kardiamobile OR AB=Kardiamobile) OR (TI="Pulse oximeter*" OR AB="Pulse oximeter*") OR (TI=pulse-ox* OR AB=pulse-ox*) OR (TI=Cuffless OR AB=Cuffless) OR (TI="Empatica E4" OR AB="Empatica E4") OR (TI="Finger probe*" OR AB="Finger probe*") OR (TI=Fitbit OR AB=Fitbit) OR (TI="Smart watch*" OR AB="Smart watch*") OR (TI=smartwatch* OR AB=smartwatch*) OR (TI=Xiaomi OR AB=Xiaomi) OR (TI=Biovotion OR AB=Biovotion) OR (TI="Basis peak" OR AB="Basis peak") OR (TI="Polar M600" OR AB="Polar M600") OR (TI=Misfit OR AB=Misfit) OR (TI=Withings OR AB=Withings) OR (TI=Mio OR AB=Mio) OR (TI=Samsung OR AB=Samsung) OR (TI=PulseON OR AB=PulseON) OR (TI=TomTom OR AB=TomTom) OR (TI=Empatica OR AB=Empatica) OR (TI=Optoelectronic OR AB=Optoelectronic) OR (TI="reflection index*" OR AB="reflection index*") OR (TI="stiffness index*" OR AB="stiffness index*")))

**ClinicalTrials.gov - 185 on 4/15/22**

Other Terms: Photoplethysmography

**DRAFT SEARCH METHODS:**

The following databases were searched from inception to March 15, 2022 in order to identify relevant articles, trials, or meeting abstracts describing photoplethysmography and skin of color: PubMed.gov, Elsevier Embase (including Embase Classic), Elsevier Scopus, Web of Science Core Collection (SCI-EXPANDED; SSCI; A&HCI; CPCI-S; CPCI-SSH; BKCI-S; BKCI-SSH; ESCI; CCR-EXPANDED), CINAHL Complete, GoogleScholar, and ClinicalTrials.gov. Each search utilized controlled vocabulary whenever possible in combination with relevant keywords. No limits were applied to the search. A set of sentinel articles were identified before the search process and were used to generate search terms and test the effectiveness of the strategies in each database. Reference tracking was performed on highly relevant articles. Original search strategies were developed in Pubmed and translated as appropriate to the other databases using the Systematic Review Accelerator Polyglot tool. Citations were deduplicated using DistillerSR's deduplication tools.

## **Data Extraction**

We developed a comprehensive data extraction form based on the guidelines from the

Cochrane Handbook for Systematic Reviews of Interventions^170^. A consort chart of the literature search is shown in Figure 1. Studies that met inclusion criteria were included in the meta-analysis (a total of 27 original studies). Two reviewers (BWN, SS) independently extracted data from published reports and pulled participant-level data from Bent et al. supplementary materials.^171^ A third reviewer (MB, HG) adjudicated any differences between the initial two reviewers and resolved any disagreements by checking manuscripts. All articles were verified with a quality check by the first author. A standardized data extraction system was developed to extract the study characteristics (Authors, Year, Sample size, study setting (inpatient, outpatient, research setting- lab, research setting- real world, other), Skin tone measurements (Skin pigmentation measurement method), sample size by skin tone categorized as light, medium, dark, race^172^ (sample size by race groups), ethnicity (sample size by ethnicity groups), description of study population (medical vs. healthy), device type (medical, non-medical), reference device (electrocardiogram, arterial blood gas), measurement method (transmittance, reflectance), device location (finger, wrist, ear, forehead, chest, other (If no location was identified in a paper, “other” was selected as the location). and results (mean bias with SD, SE , 95% CI for race, ethnicity and skin tone). For one paper, we used open access to data in supplementary materials to calculate mean bias and SE by each skin pigmentation category. We used skin tone, race, and ethnicity to categorize participants into light, medium, and dark skin pigmentation categories that were defined by race, ethnicity and skin tone.

## **Open Code and Data**

Open code and data available at osf.io/qngmz/.

## **Additional Details on Method for Statistical Analysis**

Following Shi. et. al. we applied following methods to adjust standard deviation of bias, when repeated measures were taken within a study.

- adjusted SD^2^= reported SD^2^ * [(the total number of repeated measures-1) / (the total number of repeated measures - the average number of replications per participant)]

Subsequently the sampling variance of mean bias for such a scenario is taken as (adjusted SD^2^ / the number of participants).

In order to pool SD across studies, adopting Tipton and Shuster 2017, we used the below transformation to stabilize and normalize the estimates of SD for each study, before feeding into the CHE models.

- point estimate of log(adjusted σ^2^) = log(adjusted SD^2^) + 1 / (the number of participants-1)
- sampling variance of log(adjusted σ^2^) = 2 / (the number of participants - 1)

When a study has only 1 subject, the study was excluded from CHE modeling to pool SD. Antilog was taken to report the pooled SD from the CHE model.

# **References**

1. Shi C, Goodall M, Dumville J, et al. The accuracy of pulse oximetry in measuring oxygen saturation by levels of skin pigmentation: a systematic review and meta-analysis. *BMC Med*. 2022;20(1):267. doi:10.1186/s12916-022-02452-8

2. Banerjee S, Banerjee R, Zhuang L, Persen K. WRIST WORNE WEARABLE FOR DETECTING ATRIAL FIBRILLATION. *J Am Coll Cardiol*. 2020;75(11):3561.

3. Bhoyar A, Furmston A, Daniels J, et al. Pulse oximetry as a screening tool for detecting congenital heart defects in the West Midlands, United Kingdom - An interim analysis. *Cardiol Young*. 2010;20:152.

4. Blanchet MA, Mercier G, Bouchard PA, Rousseau E, Lellouche F. Accuracy of pulse oximetry (SpO2) with different oximeters. Oxygap study. *Intensive Care Med Exp*. 2021;9(SUPPL 1). https://www.embase.com/search/results?subaction=viewrecord&id=L636288423&from=export http://dx.doi.org/10.1186/s40635-021-00413-8

5. Foglia E, Whyte R, Chaudhary A, et al. Accuracy and precision of pulse oximetry in hypoxemic infants. *Eur J Pediatr*. 2016;175(11):1584-1585.

6. Kapur V, Wilsdon T, Au DH, et al. Resting oxygen saturation was lower in obese elderly people in the cardiovascular health study. *Am J Respir Crit Care Med*. 2011;183(1). https://www.embase.com/search/results?subaction=viewrecord&id=L70847994&from=export

7. Nickel A, Jiang S, Napolitano N, et al. Impact of skin color on accuracy of capillary refill time measurement by pulse oximeter. *Circulation*. 2018;138. https://www.embase.com/search/results?subaction=viewrecord&id=L626956182&from=export

8. Sathiyakumar A, Pitrowsky M, Davidson M, et al. Developing a predictive model for assessing PAO2/FIO2 using SAO2/FIO2 in critically ill patients. *Crit Care Med*. 2011;39:138.

9. Vybornova A, Wurzner-Ghajarzadeh A, Polychronopoulou E, Fallet S, Sola J, Wuerzner G. Blood pressure from the optical aktiia bracelet: A one month validation study using an adapted iso81060-2 protocol. *J Hypertens*. 2021;39(SUPPL 1):e133-e134.

10. Cui W, Ostrander LE, Lee BY. In vivo reflectance of blood and tissue as a function of light wavelength. *IEEE Trans Biomed Eng*. 1990;37(6):632-639. doi:10.1109/10.55667

11. Fallow BA, Tarumi T, Tanaka H. Influence of skin type and wavelength on light wave reflectance. *J Clin Monit Comput*. 2013;27(3):313-317. doi:10.1007/s10877-013-9436-7

12. Mark N, Lyubin A, Gerasi R, et al. Comparison of the Effects of Motion and Environment Conditions on Accuracy of Handheld and Finger-Based Pulse Oximeters. *Mil Med*. 2021;186(Supplement_1):465-472. doi:10.1093/milmed/usaa314

13. Avant MG, Lowe N, Torres Jr A. Comparison of accuracy and signal consistency of two reusable pulse oximeter probes in critically ill children. *Respir Care*. 1997;42(7):698-704.

14. Arnold CG, Walker JR, Young S, Brady MF. 100EMF Ankle-Brachial Index Measured Using Pulse Oximeter Plethysmograph Waveform and Automated Blood Pressure Device to Assess Lower Extremity Vascular Integrity. *Ann Emerg Med*. 2019;74(4):S40.

15. Gillinov S, Etiwy M, Wang R, et al. Variable Accuracy of Wearable Heart Rate Monitors during Aerobic Exercise. *Med Sci Sports Exerc*. 2017;49(8):1697-1703. doi:10.1249/MSS.0000000000001284

16. Huynh P, Shan R, Osuji N, et al. Heart Rate Measurements in Patients with Obstructive Sleep Apnea and Atrial Fibrillation: Prospective Pilot Study Assessing Apple Watch’s Agreement With Telemetry Data. *JMIR Cardio*. 2021;5(1):e18050. doi:10.2196/18050

17. Reddy RK, Pooni R, Zaharieva DP, et al. Accuracy of Wrist-Worn Activity Monitors During Common Daily Physical Activities and Types of Structured Exercise: Evaluation Study. *JMIR MHealth UHealth*. 2018;6(12):e10338. doi:10.2196/10338

18. Seshadri DR, Bittel B, Browsky D, et al. Accuracy of the Apple Watch 4 to Measure Heart Rate in Patients With Atrial Fibrillation. *IEEE J Transl Eng Health Med*. 2020;8:1-4. doi:10.1109/JTEHM.2019.2950397

19. Hochstadt A, Havakuk O, Chorin E, et al. Continuous heart rhythm monitoring using mobile photoplethysmography in ambulatory patients. *J Electrocardiol*. 2020;60:138-141. doi:10.1016/j.jelectrocard.2020.04.017

20. Webster DE, Tummalacherla M, Higgins M, et al. Smartphone-Based VO2max Measurement With Heart Snapshot in Clinical and Real-world Settings With a Diverse Population: Validation Study. *JMIR MHealth UHealth*. 2021;9(6):e26006. doi:10.2196/26006

21. Giggins OM, Doyle J, Smith S, Crabtree DR, Fraser M. Measurement of Heart Rate Using the Withings ScanWatch Device During Free-living Activities: Validation Study. *JMIR Form Res*. 2022;6(9):e34280. doi:10.2196/34280

22. Støve MP, Hansen ECK. Accuracy of the Apple Watch Series 6 and the Whoop Band 3.0 for assessing heart rate during resistance exercises. *J Sports Sci*. 2022;40(23):2639-2644. doi:10.1080/02640414.2023.2180160

23. Blondel N, Perdrix J. Hypoxemia: The limits of the pulse oximeter, especially in the case of dark skin. *Rev Med Suisse*. 2021;17(722):158.

24. Gutmann J. Evaluation of the function of the small vessels by photo-plethysmography with reflecting light. *Vasomed*. 2003;15(2):52-56.

25. Housset E. Vasomotor action of the extremities. *Rev Prat*. 1957;7(1):7-13.

26. Lee KH, Hui KP, Tan WC, Lim TK. Limitations of pulse oximetry in Singapore’s multi-ethnic society. *Clin Intensive Care*. 1992;3(2 SUPPL.):37.

27. Yan L, Hu S, Alzahrani A, Alharbi S, Blanos P. A Multi-Wavelength Opto-Electronic Patch Sensor to Effectively Detect Physiological Changes against Human Skin Types. *Biosensors*. 2017;7(4):22. doi:10.3390/bios7020022

28. Francesco Scardulla, Sijung Hu, Leonardo D’Acquisto, et al. A novel multi-wavelength procedure for blood pressure estimation using opto-physiological sensor at peripheral arteries and capillaries. In: Vol 10486. ; 2018:1048614. doi:10.1117/12.2287845

29. Mantri Y, Jokerst JV. Impact of skin tone on photoacoustic oximetry and tools to minimize bias. *Biomed Opt Express*. 2022;13(2):875. doi:10.1364/BOE.450224

30. Jang SI, Lee M, Han J, et al. A study of skin characteristics with long-term sleep restriction in Korean women in their 40s. *Skin Res Technol Off J Int Soc Bioeng Skin ISBS Int Soc Digit Imaging Skin ISDIS Int Soc Skin Imaging ISSI*. 2020;26(2):193-199. doi:10.1111/srt.12797

31. Nikseresht F, Yan R, Lew R, Liu Y, Sebastian RM, Doryab A. Detection of Racial Bias from Physiological Responses. Ahram TZF CS, ed. *AHFE Conf Usability User Exp Hum Factors Wearable Technol Hum Factors Virtual Environ Game Des Hum Factors Assist Technol 2021*. 2021;275:59-66.

32. Velichkovska B, Gjoreski H, Denkovski D, et al. Vital signs as a source of racial bias. *medRxiv*. 2022;((Velichkovska B., bojanav@feit.ukim.edu.mk; Gjoreski H., hristijang@feit.ukim.edu.mk; Denkovski D., danield@feit.ukim.edu.mk; Kalendar M., marijaka@feit.ukim.edu.mk) Ss. Cyril and Methodius University, Faculty of Electrical Engineering and Information Technologies, Skopje, Macedonia). https://www.embase.com/search/results?subaction=viewrecord&id=L2017166180&from=export http://dx.doi.org/10.1101/2022.02.03.22270291

33. Mukherjee R, Ghosh S, Gupta B, Chakravarty T. A Universal Noninvasive Continuous Blood Pressure Measurement System for Remote Healthcare Monitoring. *Telemed E-Health*. 2018;24(10):803-810. doi:10.1089/tmj.2017.0257

34. Murphy SM, Omar S. The Clinical Utility of Noninvasive Pulse Co-oximetry Hemoglobin Measurements in Dark-Skinned Critically Ill Patients. *Anesth Analg*. 2018;126(5):1519-1526. doi:10.1213/ANE.0000000000002721

35. Gibney MA, Arce CH, Byron KJ, Hirsch LJ. Skin and subcutaneous adipose layer thickness in adults with diabetes at sites used for insulin injections: implications for needle length recommendations. *Curr Med Res Opin*. 2010;26(6):1519-1530. doi:10.1185/03007995.2010.481203

36. Huang N, Zhou M, Bian D, et al. Novel Continuous Respiratory Rate Monitoring Using an Armband Wearable Sensor. In: *2021 43rd Annual International Conference of the IEEE Engineering in Medicine & Biology Society (EMBC)*. IEEE; 2021:7470-7475. doi:10.1109/EMBC46164.2021.9630025

37. Nachman D, Eisenkraft A, Goldstein N, et al. Influence of Sex, BMI, and Skin Color on the Accuracy of Non-Invasive Cuffless Photoplethysmography-Based Blood Pressure Measurements. *Front Physiol*. 2022;13:911544. doi:10.3389/fphys.2022.911544

38. Hussain SA. Pulse oximetry interference in bronze baby syndrome. *J Perinatol*. 2009;29(12):828-829. doi:10.1038/jp.2009.35

39. Kashimutt S. Black henna and pulse oximeter. *Anaesthesia*. 2014;69:84.

40. Bonafide CP, Xiao R, Brady PW, et al. Prevalence of Continuous Pulse Oximetry Monitoring in Hospitalized Children With Bronchiolitis Not Requiring Supplemental Oxygen. *JAMA*. 2020;323(15):1467. doi:10.1001/jama.2020.2998

41. Lipchak D, Chupov A. Sensorex: The Challenges for Engineering Implementation of Low-Cost Non-Invasive Pulse Oximeter Applicable to Diverse Patient Population. *22nd IEEE Int Conf Young Prof Electron Devices Mater EDM 2021*. 2021;2021-June:106-109.

42. Ojeda CM, Dela Cruz JC. Analysis on the Optical Response of Ldr and Phototransistor in Photoplethysmography. *Sens Bio-Sens Res*. 2020;28:100334. doi:10.1016/j.sbsr.2020.100334

43. Rahnama’i MS, Geilen RP, Singhi S, Van Den Akker M, Chavannes NH. Which clinical signs and symptoms predict hypoxemia in acute childhood asthma? *Indian J Pediatr*. 2006;73(9):771-775.

44. Rodriguez-Quinonez JC, Sergiyenko O, Basaca-Preciado LC, Rivas-Lopez M. Analysis of laser light reflectance on the human skin for optoelectronic devices. *25th IEEE Photonics Conf IPC 2012*. Published online 2012:80-81.

45. Sartor O, Morris MJ, Kraus BJ. Racial Bias in Pulse Oximetry Measurement (vol 383, pg 2477, 2020). *N Engl J Med*. 385(26):2496-2496.

46. Vandeput JJ, Tanner JC, Beckers R. Photoelectric Plethysmography in Monitoring Skin Circulation: *South Med J*. 1990;83(5):533-537. doi:10.1097/00007611-199005000-00012

47. Bangash MN, Hodson J, Evison F, et al. Impact of ethnicity on the accuracy of measurements of oxygen saturations: A retrospective observational cohort study. *eClinicalMedicine*. 2022;48:101428. doi:10.1016/j.eclinm.2022.101428

48. Henry NR, Hanson AC, Schulte PJ, et al. Disparities in Hypoxemia Detection by Pulse Oximetry Across Self-Identified Racial Groups and Associations With Clinical Outcomes*. *Crit Care Med*. 2022;50(2):204-211. doi:10.1097/CCM.0000000000005394

49. Hermand E, Cassirame J, Ennequin G, Hue O. Validation of a Photoplethysmographic Heart Rate Monitor: Polar OH1. *Int J Sports Med*. 2019;40(07):462-467. doi:10.1055/a-0875-4033

50. Lee KH, Hui KP, Tan WC, Lim TK. Factors influencing pulse oximetry as compared to functional arterial saturation in multi-ethnic Singapore. *Singapore Med J*. 1993;34(5):385-387.

51. Puranen A, Halkola T, Kirkeby O, Vehkaoja A. Effect of skin tone and activity on the performance of wrist-worn optical beat-to-beat heart rate monitoring. In: *2020 IEEE SENSORS*. IEEE; 2020:1-4. doi:10.1109/SENSORS47125.2020.9278523

52. Ray I, Liaqat D, Gabel M, de Lara E. Skin tone, Confidence, and Data Quality of Heart Rate Sensing in WearOS Smartwatches. In: *2021 IEEE International Conference on Pervasive Computing and Communications Workshops and Other Affiliated Events (PerCom Workshops)*. IEEE; 2021:213-219. doi:10.1109/PerComWorkshops51409.2021.9431120

53. Ries AL, Prewitt LM, Johnson JJ. Skin color and ear oximetry. *Chest*. 1989;96(2):287-290. doi:10.1378/chest.96.2.287

54. Shcherbina A, Mattsson C, Waggott D, et al. Accuracy in Wrist-Worn, Sensor-Based Measurements of Heart Rate and Energy Expenditure in a Diverse Cohort. *J Pers Med*. 2017;7(2):3. doi:10.3390/jpm7020003

55. Sjoding MW, Dickson RP, Iwashyna TJ, Gay SE, Valley TS. Racial Bias in Pulse Oximetry Measurement. *N Engl J Med*. 2020;383(25):2477-2478. doi:10.1056/NEJMc2029240

56. Wiles MD, El‐Nayal A, Elton G, et al. Effect of patient ethnicity on the accuracy of peripheral pulse oximetry in patients with COVID‐19 pneumonitis requiring mechanical ventilation. *Anaesthesia*. 2022;77(4):489-491. doi:10.1111/anae.15656

57. Wong AKI, Charpignon M, Kim H, et al. Analysis of Discrepancies Between Pulse Oximetry and Arterial Oxygen Saturation Measurements by Race and Ethnicity and Association With Organ Dysfunction and Mortality. *JAMA Netw Open*. 2021;4(11):e2131674. doi:10.1001/jamanetworkopen.2021.31674

58. Brooks JC, Raman S, Gibbons K, et al. *Transcutaneous Oxygen Saturation Accuracy in Critically Ill Children*. In Review; 2020. doi:10.21203/rs.2.21938/v1

59. Cecil WT, Thorpe KJ, Fibuch EE, Tuohy GF. A clinical evaluation of the accuracy of the Nellcor N-100 and Ohmeda 3700 pulse oximeters. *J Clin Monit*. 1988;4(1):31-36. doi:10.1007/BF01618105

60. Escourrou PJ, Delaperche MF, Visseaux A. Reliability of pulse oximetry during exercise in pulmonary patients. *Chest*. 1990;97(3):635-638. doi:10.1378/chest.97.3.635

61. Espinosa HG, Thiel DV, Sorell M, Rowlands D. Can We Trust Inertial and Heart Rate Sensor Data from an APPLE Watch Device? In: *The 13th Conference of the International Sports Engineering Association*. MDPI; 2020:128. doi:10.3390/proceedings2020049128

62. Etiwy M, Akhrass Z, Gillinov L, et al. Accuracy of wearable heart rate monitors in cardiac rehabilitation. *Cardiovasc Diagn Ther*. 2019;9(3):262-271. doi:10.21037/cdt.2019.04.08

63. Gabrielczyk MR, Buist RJ. Pulse oximetry and postoperative hypothermia. An evaluation of the Nellcor N-100 in a cardiac surgical intensive care unit. *Anaesthesia*. 1988;43(5):402-404. doi:10.1111/j.1365-2044.1988.tb09025.x

64. Harris BU, Stewart S, Verma A, et al. Accuracy of a portable pulse oximeter in monitoring hypoxemic infants with cyanotic heart disease. *Cardiol Young*. 2019;29(8):1025-1029. doi:10.1017/S1047951119001355

65. Harris BU, Char DS, Feinstein JA, Verma A, Shiboski SC, Ramamoorthy C. Accuracy of Pulse Oximeters Intended for Hypoxemic Pediatric Patients. *Pediatr Crit Care Med J Soc Crit Care Med World Fed Pediatr Intensive Crit Care Soc*. 2016;17(4):315-320. doi:10.1097/PCC.0000000000000660

66. Harskamp RE, Bekker L, Himmelreich JCL, et al. Performance of popular pulse oximeters compared with simultaneous arterial oxygen saturation or clinical-grade pulse oximetry: a cross-sectional validation study in intensive care patients. *BMJ Open Respir Res*. 2021;8(1):e000939. doi:10.1136/bmjresp-2021-000939

67. Horton JF, Stergiou P, Fung TS, Katz L. Comparison of Polar M600 Optical Heart Rate and ECG Heart Rate during Exercise. *Med Sci Sports Exerc*. 2017;49(12):2600-2607. doi:10.1249/MSS.0000000000001388

68. Menghini L, Gianfranchi E, Cellini N, Patron E, Tagliabue M, Sarlo M. Stressing the accuracy: Wrist‐worn wearable sensor validation over different conditions. *Psychophysiology*. 2019;56(11):e13441. doi:10.1111/psyp.13441

69. Pasadyn SR, Soudan M, Gillinov M, et al. Accuracy of commercially available heart rate monitors in athletes: a prospective study. *Cardiovasc Diagn Ther*. 2019;9(4):379-385. doi:10.21037/cdt.2019.06.05

70. Ries AL, Farrow JT, Clausen JL. Accuracy of two ear oximeters at rest and during exercise in pulmonary patients. *Am Rev Respir Dis*. 1985;132(3):685-689. doi:10.1164/arrd.1985.132.3.685

71. Ross PA, Newth CJL, Khemani RG. Accuracy of pulse oximetry in children. *Pediatrics*. 2014;133(1):22-29.

72. Schallom M, Prentice D, Sona C, Arroyo C, Mazuski J. Comparison of nasal and forehead oximetry accuracy and pressure injury in critically ill patients. *Heart Lung J Crit Care*. 2018;47(2):93-99. doi:10.1016/j.hrtlng.2017.12.002

73. Smyth RJ, D’Urzo AD, Slutsky AS, Galko BM, Rebuck AS. Ear oximetry during combined hypoxia and exercise. *J Appl Physiol Bethesda Md 1985*. 1986;60(2):716-719. doi:10.1152/jappl.1986.60.2.716

74. Spierer DK, Rosen Z, Litman LL, Fujii K. Validation of photoplethysmography as a method to detect heart rate during rest and exercise. *J Med Eng Technol*. 2015;39(5):264-271. doi:10.3109/03091902.2015.1047536

75. Stewart KG, Rowbottom SJ. Inaccuracy of pulse oximetry in patients with severe tricuspid regurgitation. *Anaesthesia*. 1991;46(8):668-670. doi:10.1111/j.1365-2044.1991.tb09720.x

76. Valbuena VSM, Seelye S, Sjoding MW, et al. Racial bias and reproducibility in pulse oximetry among medical and surgical inpatients in general care in the Veterans Health Administration 2013-19: multicenter, retrospective cohort study. *BMJ*. Published online July 6, 2022:e069775. doi:10.1136/bmj-2021-069775

77. Wallen MP, Gomersall SR, Keating SE, Wisløff U, Coombes JS. Accuracy of Heart Rate Watches: Implications for Weight Management. *PloS One*. 2016;11(5):e0154420. doi:10.1371/journal.pone.0154420

78. Wang YT, Poh SC. Noninvasive oximetry in pigmented patients. *Ann Acad Med Singapore*. 1985;14(3):427-429.

79. Pipek LZ, Nascimento RFV, Acencio MMP, Teixeira LR. Comparison of SpO2 and heart rate values on Apple Watch and conventional commercial oximeters devices in patients with lung disease. *Sci Rep*. 2021;11(1):18901. doi:10.1038/s41598-021-98453-3

80. Harrison DK, Greenidge AR, Landis RC. Skin SO₂ measurement using visible lightguide spectrophotometry in a black population: a feasibility study. *Adv Exp Med Biol*. 2011;701:277-282.

81. Ralston AC, Webb RK, Runciman WB. Potential errors in pulse oximetry. III: Effects of interference, dyes, dyshaemoglobins and other pigments. *Anaesthesia*. 1991;46(4):291-295.

82. Adler JN, Hughes LA, Vtvilecchia R, Jr. CAC. Effect of Skin Pigmentation on Pulse Oximetry Accuracy in the Emergency Department. *Acad Emerg Med*. 1998;5(10):965-970. doi:10.1111/j.1553-2712.1998.tb02772.x

83. Bickler PE, Feiner JR, Severinghaus JW. Effects of Skin Pigmentation on Pulse Oximeter Accuracy at Low Saturation. *Anesthesiology*. 2005;102(4):715-719. doi:10.1097/00000542-200504000-00004

84. Bothma PA, Joynt GM, Lipman J, et al. Accuracy of pulse oximetry in pigmented patients. *South Afr Med J Suid-Afr Tydskr Vir Geneeskd*. 1996;86(5 Suppl):594-596.

85. Ebmeier SJ, Barker M, Bacon M, et al. A Two Centre Observational Study of Simultaneous Pulse Oximetry and Arterial Oxygen Saturation Recordings in Intensive Care Unit Patients. *Anaesth Intensive Care*. 2018;46(3):297-303. doi:10.1177/0310057X1804600307

86. Feiner JR, Severinghaus JW, Bickler PE. Dark Skin Decreases the Accuracy of Pulse Oximeters at Low Oxygen Saturation: The Effects of Oximeter Probe Type and Gender. *Anesth Analg*. 2007;105(6):S18-S23. doi:10.1213/01.ane.0000285988.35174.d9

87. Foglia EE, Whyte RK, Chaudhary A, et al. The Effect of Skin Pigmentation on the Accuracy of Pulse Oximetry in Infants with Hypoxemia. *J Pediatr*. 2017;182:375-377.e2. doi:10.1016/j.jpeds.2016.11.043

88. Hinkelbein J, Genzwuerker HV, Sogl R, Fiedler F. Effect of nail polish on oxygen saturation determined by pulse oximetry in critically ill patients. *Resuscitation*. 2007;72(1):82-91. doi:10.1016/j.resuscitation.2006.06.024

89. Hinkelbein J, Koehler H, Genzwuerker HV, Fiedler F. Artificial acrylic finger nails may alter pulse oximetry measurement. *Resuscitation*. 2007;74(1):75-82. doi:10.1016/j.resuscitation.2006.11.018

90. Jubran A, Tobin MJ. Reliability of pulse oximetry in titrating supplemental oxygen therapy in ventilator-dependent patients. *Chest*. 1990;97(6):1420-1425. doi:10.1378/chest.97.6.1420

91. Vesoulis Z, Tims A, Lodhi H, Lalos N, Whitehead H. Racial discrepancy in pulse oximeter accuracy in preterm infants. *J Perinatol*. 2022;42(1):79-85. doi:10.1038/s41372-021-01230-3

92. Wiles MD, El-Nayal A, Elton G, et al. The effect of patient ethnicity on the accuracy of peripheral pulse oximetry in patients with COVID-19 pneumonitis: a single-centre, retrospective analysis. *Anaesthesia*. 77(2):143-152.

93. Zeballos RJ, Weisman IM. Reliability of noninvasive oximetry in black subjects during exercise and hypoxia. *Am Rev Respir Dis*. 1991;144(6):1240-1244. doi:10.1164/ajrccm/144.6.1240

94. Incorrect Numbers in eTable and Text. *JAMA Netw Open*. 2022;5(2):e221210. doi:10.1001/jamanetworkopen.2022.1210

95. Erratum: Racial Bias in Pulse Oximetry Measurement (N Engl J Med (2020) 383 (2477-2478) DOI: 10.1056/NEJMc2029240). *N Engl J Med*. 2021;385(26):2496.

96. Bent B, Enache OM, Goldstein B, Kibbe W, Dunn JP. Reply: Matters Arising ‘Investigating sources of inaccuracy in wearable optical heart rate sensors.’ *Npj Digit Med*. 2021;4(1). https://www.scopus.com/inward/record.uri?eid=2-s2.0-85101924150&doi=10.1038%2fs41746-021-00409-4&partnerID=40&md5=d60f4c8a073b97b6d8c6b3006cb30951

97. Whitehead-Clarke T. More on Racial Bias in Pulse Oximetry Measurement. *N Engl J Med*. 384(13):1278.

98. Okunlola OE, Lipnick MS, Batchelder PB, Bernstein M, Feiner JR, Bickler PE. Pulse Oximeter Performance, Racial Inequity, and the Work Ahead. *Respir Care*. 2022;67(2):252-257. doi:10.4187/respcare.09795

99. Shi C, Goodall M, Dumville J, et al. The effects of skin pigmentation on the accuracy of pulse oximetry in measuring oxygen saturation: a systematic review and meta-analysis. *medRxiv*. 2022;((Shi C., chunhu.shi@manchester.ac.uk; Dumville J.; Norman G.; Cullum N.) School of Health Sciences, Faculty of Biology, Medicine and Health, Manchester Academic Health Science Centre, University of Manchester, Manchester, United Kingdom). https://www.embase.com/search/results?subaction=viewrecord&id=L2017166650&from=export http://dx.doi.org/10.1101/2022.02.16.22271062

100. Garrett A, Kim B, Sie EJ, et al. Simultaneous photoplethysmography and blood flow measurements towards the estimation of blood pressure using speckle contrast optical spectroscopy. *Biomed Opt Express*. 2023;14(4):1594. doi:10.1364/BOE.482740

101. Bai Y, Hibbing P, Mantis C, Welk GJ. Comparative evaluation of heart rate-based monitors: Apple Watch vs Fitbit Charge HR. *J Sports Sci*. 2018;36(15):1734-1741. doi:10.1080/02640414.2017.1412235

102. Bellenger C, Miller D, Halson S, Roach G, Sargent C. Wrist-Based Photoplethysmography Assessment of Heart Rate and Heart Rate Variability: Validation of WHOOP. *Sensors*. 2021;21(10):3571. doi:10.3390/s21103571

103. Benedetti D, Olcese U, Frumento P, et al. Heart rate detection by Fitbit ChargeHR ^TM^ : A validation study versus portable polysomnography. *J Sleep Res*. 2021;30(6):e13346. doi:10.1111/jsr.13346

104. Berryhill S, Morton CJ, Dean A, et al. Effect of wearables on sleep in healthy individuals: a randomized crossover trial and validation study. *J Clin Sleep Med*. 2020;16(5):775-783. doi:10.5664/jcsm.8356

105. Boudreaux BD, Hebert EP, Hollander DB, et al. Validity of Wearable Activity Monitors during Cycling and Resistance Exercise. *Med Sci Sports Exerc*. 2018;50(3):624-633. doi:10.1249/MSS.0000000000001471

106. Cadmus-Bertram L, Gangnon R, Wirkus EJ, Thraen-Borowski KM, Gorzelitz-Liebhauser J. The Accuracy of Heart Rate Monitoring by Some Wrist-Worn Activity Trackers. *Ann Intern Med*. 2017;166(8):610. doi:10.7326/L16-0353

107. Cao R, Azimi I, Sarhaddi F, et al. Accuracy Assessment of Oura Ring Nocturnal Heart Rate and Heart Rate Variability in Comparison With Electrocardiography in Time and Frequency Domains: Comprehensive Analysis. *J Med Internet Res*. 2022;24(1):e27487. doi:10.2196/27487

108. De Zambotti M, Baker FC, Willoughby AR, et al. Measures of sleep and cardiac functioning during sleep using a multi-sensory commercially-available wristband in adolescents. *Physiol Behav*. 2016;158:143-149. doi:10.1016/j.physbeh.2016.03.006

109. Dooley EE, Golaszewski NM, Bartholomew JB. Estimating Accuracy at Exercise Intensities: A Comparative Study of Self-Monitoring Heart Rate and Physical Activity Wearable Devices. *JMIR MHealth UHealth*. 2017;5(3):e34. doi:10.2196/mhealth.7043

110. Gorny AW, Liew SJ, Tan CS, Müller-Riemenschneider F. Fitbit Charge HR Wireless Heart Rate Monitor: Validation Study Conducted Under Free-Living Conditions. *JMIR MHealth UHealth*. 2017;5(10):e157. doi:10.2196/mhealth.8233

111. Held NJ, Perrotta AS, Mueller T, Pfoh-MacDonald SJ. Agreement of the Apple Watch® and Fitbit Charge® for recording step count and heart rate when exercising in water. *Med Biol Eng Comput*. 2022;60(5):1323-1331. doi:10.1007/s11517-022-02536-w

112. Hettiarachchi IT, Hanoun S, Nahavandi D, Nahavandi S. Validation of Polar OH1 optical heart rate sensor for moderate and high intensity physical activities. Boullosa D, ed. *PLOS ONE*. 2019;14(5):e0217288. doi:10.1371/journal.pone.0217288

113. Kinnunen H, Rantanen A, Kenttä T, Koskimäki H. Feasible assessment of recovery and cardiovascular health: accuracy of nocturnal HR and HRV assessed via ring PPG in comparison to medical grade ECG. *Physiol Meas*. 2020;41(4):04NT01. doi:10.1088/1361-6579/ab840a

114. Kroll RR, Boyd JG, Maslove DM. Accuracy of a Wrist-Worn Wearable Device for Monitoring Heart Rates in Hospital Inpatients: A Prospective Observational Study. *J Med Internet Res*. 2016;18(9):e253. doi:10.2196/jmir.6025

115. Mendelson Y, Ochs BD. Noninvasive pulse oximetry utilizing skin reflectance photoplethysmography. *IEEE Trans Biomed Eng*. 1988;35(10):798-805. doi:10.1109/10.7286

116. Miller DJ, Sargent C, Roach GD. A Validation of Six Wearable Devices for Estimating Sleep, Heart Rate and Heart Rate Variability in Healthy Adults. *Sensors*. 2022;22(16):6317. doi:10.3390/s22166317

117. Muggeridge DJ, Hickson K, Davies AV, et al. Measurement of Heart Rate Using the Polar OH1 and Fitbit Charge 3 Wearable Devices in Healthy Adults During Light, Moderate, Vigorous, and Sprint-Based Exercise: Validation Study. *JMIR MHealth UHealth*. 2021;9(3):e25313. doi:10.2196/25313

118. Nuuttila OP, Korhonen E, Laukkanen J, Kyröläinen H. Validity of the Wrist-Worn Polar Vantage V2 to Measure Heart Rate and Heart Rate Variability at Rest. *Sensors*. 2021;22(1):137. doi:10.3390/s22010137

119. Renevey P, Delgado-Gonzalo R, Lemkaddem A, et al. Respiratory and cardiac monitoring at night using a wrist wearable optical system. In: *2018 40th Annual International Conference of the IEEE Engineering in Medicine and Biology Society (EMBC)*. IEEE; 2018:2861-2864. doi:10.1109/EMBC.2018.8512881

120. Sarhaddi F, Kazemi K, Azimi I, et al. A comprehensive accuracy assessment of Samsung smartwatch heart rate and heart rate variability. Mian Qaisar S, ed. *PLOS ONE*. 2022;17(12):e0268361. doi:10.1371/journal.pone.0268361

121. Schubert M, Clark A, De La Rosa A. The Polar® OH1 Optical Heart Rate Sensor is Valid during Moderate-Vigorous Exercise. *Sports Med Int Open*. 2018;02(03):E67-E70. doi:10.1055/a-0631-0920

122. Schuurmans AAT, De Looff P, Nijhof KS, et al. Validity of the Empatica E4 Wristband to Measure Heart Rate Variability (HRV) Parameters: a Comparison to Electrocardiography (ECG). *J Med Syst*. 2020;44(11):190. doi:10.1007/s10916-020-01648-w

123. Sen-Gupta E, Wright DE, Caccese JW, et al. A Pivotal Study to Validate the Performance of a Novel Wearable Sensor and System for Biometric Monitoring in Clinical and Remote Environments. *Digit Biomark*. 2019;3(1):1-13. doi:10.1159/000493642

124. Stucky B, Clark I, Azza Y, et al. Validation of Fitbit Charge 2 Sleep and Heart Rate Estimates Against Polysomnographic Measures in Shift Workers: Naturalistic Study. *J Med Internet Res*. 2021;23(10):e26476. doi:10.2196/26476

125. Wang R, Blackburn G, Desai M, et al. Accuracy of Wrist-Worn Heart Rate Monitors. *JAMA Cardiol*. 2017;2(1):104. doi:10.1001/jamacardio.2016.3340

126. List WF. [The importance of pulse oximetry for anesthesia]. *Anaesthesiol Reanim*. 1991;16(1):5-10.

127. Dyer O. Pulse oximetry may underestimate hypoxaemia in black patients, study finds. *The BMJ*. 2020;371. https://www.embase.com/search/results?subaction=viewrecord&id=L633716842&from=export http://dx.doi.org/10.1136/bmj.m4926

128. Ferrari M, Quaresima V, Scholkmann F. Pulse oximetry, racial bias and statistical bias: further improvements of pulse oximetry are necessary. *Ann Intensive Care*. 2022;12(1):19. doi:10.1186/s13613-022-00992-z

129. Holder AL, Wong AKI. The Big Consequences of Small Discrepancies: Why Racial Differences in Pulse Oximetry Errors Matter*. *Crit Care Med*. 2022;50(2):335-337. doi:10.1097/CCM.0000000000005447

130. Norton HL. Variation in pulse oximetry readings: melanin, not ethnicity, is the appropriate variable to use when investigating bias. *Anaesthesia*. 2022;77(3):354-355. doi:10.1111/anae.15620

131. Papaioannou TG, Alexandraki KI, Karamanou M, Piperi C, Tousoulis D. Association of skin autofluorescence with arterial properties: A closer look at AGE Reader and EndoPAT 2000 commercial devices. *Exp Gerontol*. 2017;98:207-208.

132. Philip KEJ, Tidswell R, McFadyen C. Racial bias in pulse oximetry: more statistical detail may help tackle the problem. *BMJ*. Published online February 2, 2021:n298. doi:10.1136/bmj.n298

133. Ploen L, Pilcher J, Beckert L, Swanney M, Beasley R. An investigation into the bias of pulse oximeters. *Respirology*. 2016;21:6.

134. Pulse Oximetry May Be Inaccurate in Patients with Darker Skin. *AJN Am J Nurs*. 2021;121(4):16-16. doi:10.1097/01.NAJ.0000742448.35686.f9

135. Mukherjee R, Ghorai SK, Gupta B, Chakravarty T. Development of a Wearable Remote Cardiac Health Monitoring with Alerting System. *Instrum Exp Tech*. 2020;63(2):273-283. doi:10.1134/S002044122002013X

136. Harrison DK, Greenidge AR, Landis RC. Skin SO2 Measurement Using Visible Lightguide Spectrophotometry in a Black Population: A Feasibility Study. In: LaManna JC, Puchowicz MA, Xu K, Harrison DK, Bruley DF, eds. *Oxygen Transport to Tissue XXXII*. Vol 701. Advances in Experimental Medicine and Biology. Springer US; 2011:277-282. doi:10.1007/978-1-4419-7756-4_37

137. Królak A, Biskupiak J. Analysis of Amped Heart Rate Sensor Performance Under the Influence of Various External Factors and User Characteristics. In: Korbicz J, Maniewski R, Patan K, Kowal M, eds. *Current Trends in Biomedical Engineering and Bioimages Analysis*. Vol 1033. Advances in Intelligent Systems and Computing. Springer International Publishing; 2020:3-14. doi:10.1007/978-3-030-29885-2_1

138. Sjoding M, Iwashyna TJ, Valley TS. More on Racial Bias in Pulse Oximetry Measurement. Reply. *N Engl J Med*. 384(13):1278.

139. Tobin MJ, Jubran A. Unreliable pulse oximetry in dark-skin patients: a plea for algorithm disclosure. *Ann Intensive Care*. 12(1):18.

140. Colvonen PJ. Response To: Investigating sources of inaccuracy in wearable optical heart rate sensors. *Npj Digit Med*. 2021;4(1):38. doi:10.1038/s41746-021-00408-5

141. Agache PG, Dupond AS. Recent advances in non-invasive assessment of human skin blood flow. *Acta Derm Venereol Suppl Stockh*. 1994;185:47-51.

142. AlDallal SM. Mini review: leg ulcers - a secondary complication of sickle cell disease. *Int J Gen Med*. 2019;12:279-282. doi:10.2147/IJGM.S217369

143. Fine J, Branan KL, Rodriguez AJ, et al. Sources of Inaccuracy in Photoplethysmography for Continuous Cardiovascular Monitoring. *Biosensors*. 2021;11(4):126. doi:10.3390/bios11040126

144. Hay WW. The uses, benefits, and limitations of pulse oximetry in neonatal medicine: consensus on key issues. *J Perinatol Off J Calif Perinat Assoc*. 1987;7(4):347-349.

145. Hunasikatti M. Racial bias in accuracy of pulse oximetry and its impact on assessments of hypopnea and T90 in clinical studies. *J Clin Sleep Med*. 2021;17(5):1145-1145. doi:10.5664/jcsm.9178

146. Kamal AA, Harness JB, Irving G, Mearns AJ. Skin photoplethysmography--a review. *Comput Methods Programs Biomed*. 28(4):257-269.

147. Knight MJ, Subbe CP, Inada-Kim M. Racial discrepancies in oximetry: where do we stand? *Anaesthesia*. 2022;77(2):129-131.

148. Ray D, Collins T, Woolley S, Ponnapalli P. A Review of Wearable Multi-wavelength Photoplethysmography. *IEEE Rev Biomed Eng*. Published online 2021. https://www.scopus.com/inward/record.uri?eid=2-s2.0-85118273664&doi=10.1109%2fRBME.2021.3121476&partnerID=40&md5=bf8c2dec47341ea8710f455a71fcc809

149. Taylor MB, Whitwam JG. The current status of pulse oximetry. Clinical value of continuous noninvasive oxygen saturation monitoring. *Anaesthesia*. 41(9):943-949.

150. Tobin MJ, Jubran A. Pulse oximetry, racial bias and statistical bias. *Ann Intensive Care*. 12(1):2.

151. Ajmal, Boonya-Ananta T, Rodriguez AJ, Du Le VN, Ramella-Roman JC. Monte Carlo analysis of optical heart rate sensors in commercial wearables: the effect of skin tone and obesity on the photoplethysmography (PPG) signal. *Biomed Opt Express*. 2021;12(12):7445. doi:10.1364/BOE.439893

152. Flynn KA, Ownby NB, Wang P, Calhoun BH. Modeling Energy-Aware Photoplethysmography Hardware for Personalized Health Care Applications Across Skin Phototypes. In: *2021 IEEE Biomedical Circuits and Systems Conference (BioCAS)*. IEEE; 2021:01-06. doi:10.1109/BioCAS49922.2021.9644946

153. Chatterjee S, Kyriacou P. Monte Carlo Analysis of Optical Interactions in Reflectance and Transmittance Finger Photoplethysmography. *Sensors*. 2019;19(4):789. doi:10.3390/s19040789

154. Fine J, Boonya-ananta T, Rodriguez A, Ramella-Roman J, McShane M, Coté GL. Parallelized multi-layered Monte Carlo model for evaluation of a proximal phalanx photoplethysmograph. In: Coté GL, ed. *Optical Diagnostics and Sensing XX: Toward Point-of-Care Diagnostics*. SPIE; 2020:1. doi:10.1117/12.2543590

155. Emery JR. Skin pigmentation as an influence on the accuracy of pulse oximetry. *J Perinatol Off J Calif Perinat Assoc*. 1987;7(4):329-330.

156. Baek HJ, Shin J, Cho J. The Effect of Optical Crosstalk on Accuracy of Reflectance-Type Pulse Oximeter for Mobile Healthcare. *J Heal Eng*. 2018;2018:3521738.

157. Mendelson Y, Kent JC, Shahnarian A, Welch GW, Giasi RM. Simultaneous comparison of three noninvasive oximeters in healthy volunteers. *Med Instrum*. 1987;21(3):183-188.

158. Olmo Arroyo J, Khirani S, Amaddeo A, et al. A comparison of pulse oximetry and cerebral oxygenation in children with severe sleep apnea-hypopnea syndrome: a pilot study. *J Sleep Res*. 26(6):799-808.

159. Bickler PE, Feiner JR, Rollins MD. Factors Affecting the Performance of 5 Cerebral Oximeters During Hypoxia in Healthy Volunteers. *Anesth Analg*. 2013;117(4):813-823. doi:10.1213/ANE.0b013e318297d763

160. Cahan C, Decker MJ, Hoekje PL, Strohl KP. Agreement between Noninvasive Oximetric Values for Oxygen Saturation. *Chest*. 1990;97(4):814-819. doi:10.1378/chest.97.4.814

161. Smith RN, Hofmeyr R. Perioperative comparison of the agreement between a portable fingertip pulse oximeter v. a conventional bedside pulse oximeter in adult patients (COMFORT trial). *S Afr Med J*. 2019;109(3):154. doi:10.7196/SAMJ.2019.v109i3.13633

162. Stell D, Noble JJ, Kay RH, et al. Exploring the impact of pulse oximeter selection within the COVID-19 home-use pulse oximetry pathways. *BMJ Open Respir Res*. 2022;9(1):e001159. doi:10.1136/bmjresp-2021-001159

163. Witting MD, Scharf SM. Diagnostic room-air pulse oximetry: effects of smoking, race, and sex. *Am J Emerg Med*. 2008;26(2):131-136. doi:10.1016/j.ajem.2007.04.002

164. Schröder C, Förster R, Zwahlen DR, Windisch P. The Apple Watch spO2 sensor and outliers in healthy users. *NPJ Digit Med*. 2023;6(1):63. doi:10.1038/s41746-023-00814-x

165. Ahmed N, Chowdhury A, Sinharay A, Mukhopadhyay S, Ghose A, Chakravarty T. A personalized on-line calibration for photoplethysmograph based wrist wearable sensor. *2017 Glob Wirel Summit GWS 2017*. 2018;2018-January:1-5.

166. Preejith SP, Alex A, Joseph J, Sivaprakasam M. Design, development and clinical validation of a wrist-based optical heart rate monitor. In: *2016 IEEE International Symposium on Medical Measurements and Applications (MeMeA)*. IEEE; 2016:1-6. doi:10.1109/MeMeA.2016.7533786

167. Spaccarotella C, Polimeni A, Mancuso C, Pelaia G, Esposito G, Indolfi C. Assessment of Non-Invasive Measurements of Oxygen Saturation and Heart Rate with an Apple Smartwatch: Comparison with a Standard Pulse Oximeter. *J Clin Med*. 2022;11(6):1467. doi:10.3390/jcm11061467

168. Powell R, Pattison HM, Bhoyar A, et al. Pulse oximetry screening for congenital heart defects in newborn infants: an evaluation of acceptability to mothers. *Arch Dis Child - Fetal Neonatal Ed*. 2013;98(1):F59-F63. doi:10.1136/fetalneonatal-2011-301225

169. Clark JM, Sanders S, Carter M, et al. Improving the translation of search strategies using the Polyglot Search Translator: a randomized controlled trial. *J Med Libr Assoc*. 2020;108(2). doi:10.5195/jmla.2020.834

170. Higgins JPT, Deeks JJ. Selecting studies and collecting data. In: Cochrane Handbook for Systematic Reviews of Interventions. John Wiley & Sons, Ltd; 2008:151-185. ISBN: 9780470712184

171. Bent B, Goldstein BA, Kibbe WA, Dunn JP. Investigating sources of inaccuracy in wearable optical heart rate sensors. *Npj Digit Med*. 2020;3(1):18. doi:10.1038/s41746-020-0226-6

172. Revisions to the Standards for the Classification of Federal Data on Race and Ethnicity. The White House. Accessed November 16, 2023. https://obamawhitehouse.archives.gov/omb/fedreg_1997standards
